# Parcellation-induced variation of empirical and simulated brain connectomes at group and subject levels

**DOI:** 10.1162/netn_a_00202

**Published:** 2021-08-30

**Authors:** Justin W. M. Domhof, Kyesam Jung, Simon B. Eickhoff, Oleksandr V. Popovych

**Affiliations:** Institute of Neuroscience and Medicine, Brain and Behaviour (INM-7), Research Centre Jülich, Jülich, Germany; Institute for Systems Neuroscience, Medical Faculty, Heinrich Heine University Düsseldorf, Düsseldorf, Germany; Institute of Neuroscience and Medicine, Brain and Behaviour (INM-7), Research Centre Jülich, Jülich, Germany; Institute for Systems Neuroscience, Medical Faculty, Heinrich Heine University Düsseldorf, Düsseldorf, Germany; Institute of Neuroscience and Medicine, Brain and Behaviour (INM-7), Research Centre Jülich, Jülich, Germany; Institute for Systems Neuroscience, Medical Faculty, Heinrich Heine University Düsseldorf, Düsseldorf, Germany; Institute of Neuroscience and Medicine, Brain and Behaviour (INM-7), Research Centre Jülich, Jülich, Germany; Institute for Systems Neuroscience, Medical Faculty, Heinrich Heine University Düsseldorf, Düsseldorf, Germany

**Keywords:** Parcellations, Modeling, Resting state, Graph theory, Structure-function relationship

## Abstract

Recent developments of whole-brain models have demonstrated their potential when investigating resting-state brain activity. However, it has not been systematically investigated how alternating derivations of the empirical structural and functional connectivity, serving as the model input, from MRI data influence modeling results. Here, we study the influence from one major element: the brain parcellation scheme that reduces the dimensionality of brain networks by grouping thousands of voxels into a few hundred brain regions. We show graph-theoretical statistics derived from the empirical data and modeling results exhibiting a high heterogeneity across parcellations. Furthermore, the network properties of empirical brain connectomes explain the lion’s share of the variance in the modeling results with respect to the parcellation variation. Such a clear-cut relationship is not observed at the subject-resolved level per parcellation. Finally, the graph-theoretical statistics of the simulated connectome correlate with those of the empirical functional connectivity across parcellations. However, this relation is not one-to-one, and its precision can vary between models. Our results imply that network properties of both empirical connectomes can explain the goodness-of-fit of whole-brain models to empirical data at a global group level but not at a single-subject level, which provides further insights into the personalization of whole-brain models.

## Author Summary

The structural and functional connectivities of the brain, which reflect the anatomical connections of axonal bundles and the amount of coactivation between brain regions, respectively, only weakly correlate with each other. In order to enhance and investigate this relationship, large-scale whole-brain dynamical models were involved in this branch of research. However, how the definitions of the brain regions parcellated according to a so-called brain atlas influence these models has so far not been systematically assessed. In this article, we show that this influence can be large, and link group-averaged, atlas-induced deviations to network properties extracted from both types of connectivity. Additionally, we demonstrate that the same association does not apply to subject-specific variations. These results may contribute to the further personalization of the whole-brain models.

## INTRODUCTION

The **structure-function relationship** in the human brain has been a topic of interest in many neuroimaging studies (Suárez, Markello, Betzel, & Misic, 2020). Here, the **structural connectivity** (SC) and **functional connectivity** (FC), which reflect the physical connections and patterns of synchronized coactivation throughout the brain, respectively, do not exhibit a perfect association (Honey et al., 2009). One effort to close this gap in the structure-function relationship involves the employment of **dynamical whole-brain models** that use SC as prior knowledge to simulate resting-state brain activity (Honey et al., 2009). These models indeed successfully explain an additional amount of variance beyond the direct comparison of SC and FC (Honey et al., 2009). They also demonstrate that the brain at rest operates at a state of maximal metastability (Deco, Kringelbach, Jirsa, & Ritter, 2017). Other studies even suggested that the vast parameter space of the models can be exploited to reproduce resting-state brain activity on a personalized level (Ritter, Schirner, McIntosh, & Jirsa, 2013; Sanz-Leon, Knock, Spiegler, & Jirsa, 2015; Zimmermann et al., 2018).

Throughout the past decade, the workflow associated with dynamical whole-brain models investigating resting-state brain activity has matured (Bansal, Nakuci, & Muldoon, 2018; Deco, Jirsa, & McIntosh, 2011; Popovych, Manos, Hoffstaedter, & Eickhoff, 2019). When these models are derived and validated using magnetic resonance imaging (MRI) data, region-based SC and FC are typically calculated from diffusion-weighted MRI (dwMRI) and functional MRI (fMRI) sequences, respectively, so that the computations remain tractable (Bandettini, Wong, Hinks, Tikofsky, & Hyde, 1992; Kwong et al., 1992; Ogawa et al., 1992; Popovych et al., 2019; Yeh, Jones, Liang, Descoteaux, & Connelly, 2021). The reconstruction of these connectomes requires the use of a so-called **brain parcellation or brain atlas**, which describes which voxels should be included in which brain region. Over the years, many brain atlases have been constructed upon conceptually distinct underpinnings, where each of these methodologies incorporates its own biological knowledge and assumptions (e.g., the number of parcels or **granularity**) into the parcellation (Amunts & Zilles, 2015; Eickhoff, Constable, & Yeo, 2018; Eickhoff, Yeo, & Genon, 2018).

Because region-based SC and FC are reconstructed on the basis of a particular brain parcellation, it to a large extent determines the SC and FC matrices. The used brain parcellation may thus exert a substantial influence on the results of region-based neuroimaging studies. Earlier works examined the influence of parcellations on graph-theoretical measures derived from region-based SC and FC (Wang et al., 2009; Zalesky et al., 2010) and on direct SC-FC comparisons (Messé, 2020). The impact of the granularity of a brain atlas on modeling results was also investigated for the Desikan-Killiany atlas (Desikan et al., 2006) and variations of it, wherein the brain regions were split into a number of smaller subregions (Proix et al., 2016). Nevertheless, a systematic investigation of the influence of the brain parcellation is, to the best of our knowledge, currently lacking when it comes to dynamical whole-brain models replicating resting-state brain activity.

**Figure 1.**
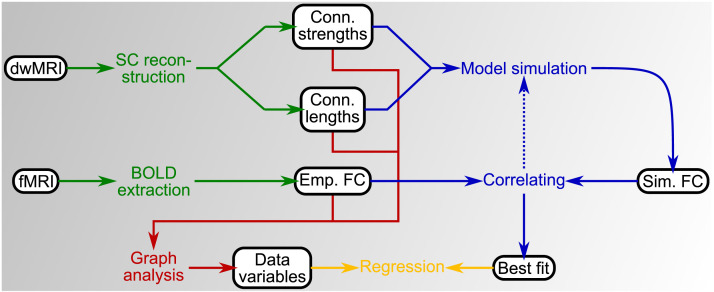
Summary of the methods used in this study. Connectome extraction (green) comprises the construction of the empirical structural (SC) and functional connectivity (FC) from the diffusion-weighted (dwMRI) and functional magnetic resonance imaging (fMRI) data, respectively. Both connectomes serve as input for the modeling stage (blue), where the model parameters are optimized to maximize the correlation between simulated and empirical data (dotted arrow). Graph-theoretical metrics were extracted from the empirical and simulated connectomes (red) and regressed with the model fitting results (orange).

Here, we investigate this influence by using the methodology outlined in Figure 1. We first extracted the SCs and FCs, henceforth referred to as the **empirical SCs** and **empirical FCs**, respectively, from the MRI data of 200 healthy subjects using 19 freely available state-of-the-art brain parcellations (Figure 1, green). We constructed the models corresponding to the SC and two qualitatively different models for the local dynamics of individual brain regions that were based on phase oscillators and a neural mass model (Figure 1, blue). By comparing between the two models, we could evaluate whether any observed effects were model-dependent. The resting-state brain activity was individually simulated for every combination of parcellation, model, and subject. Then FCs were derived from the simulated brain activity, which will henceforth be referred to as **simulated FCs**. The correlations between the simulated and empirical FCs were calculated and maximized through model parameter variations to quantify how well the models could reproduce the empirical FCs (Figure 1, blue). Finally, we compared the maximized correlations or **goodness-of-fits** with graph-theoretical measures calculated from the empirical SC and FC (Figure 1, red and orange), so that any observation regarding the modeling results could be interpreted in terms of the properties of the empirical networks used to construct and validate our models.

We found large deviations in the goodness-of-fit as brain parcellations vary. In addition, most of the group-averaged interparcellation variance in the goodness-of-fit could be attributed to variations in the graph-theoretical metrics. Such a well-pronounced relationship was practically absent when we considered within-parcellation, interindividual differences. Finally, we show that the models (inaccurately) map the empirical SC to a simulated functional network that has similar network properties as the empirical FC. Our investigation therefore illustrates how the results produced by a dynamical whole-brain modeling workflow are influenced by the brain parcellation, and reveals some of its current limitations and open issues. The reported results are relevant when considering personalized models of resting-state brain dynamics in the framework of precision medicine.

## MATERIALS AND METHODS

In this study, we systematically investigated the influence of the brain atlas on the validation of dynamical whole-brain models by using the methodology outlined in Figure 1. First, we extracted the empirical SC and FC matrices corresponding to a particular parcellation from the dwMRI and fMRI data, respectively (Figure 1, green). The result of the empirical SC reconstruction comprised two matrices: one with the number of streamlines and one with the average length of the streamlines between each pair of brain regions, which are referred to as the actual structural connectivities (SCs) and the path lengths (PLs), respectively. The empirical FC matrix contained the Pearson correlation coefficients across the BOLD response time series extracted from the fMRI data.

Subsequently, the empirical SC and PL matrices were fed to the model as prior knowledge, while the empirical FC matrix was compared with the simulated FC matrix produced by the model simulations (Figure 1, blue). Two models (a phase oscillator and a neural mass model) were used for the acquisition of the simulation results, and we simulated both models for a broad range of global parameter settings to maximize the fit between the empirical and simulated FC. We also extracted some graph-theoretical metrics from the empirical SC and PL and the empirical and simulated FC matrices (Figure 1, red). To be specific, we determined the degree distribution and the modularity of the empirical SC and both types of FC to characterize their centrality and segregation, respectively. In addition, we calculated the closeness centrality distribution and the global efficiency of the PL matrix as representations of its centrality and integration, respectively. The latter two metrics calculated from the PL matrix are based on the streamline path lengths between brain regions and allow a natural interpretability of the obtained quantities (see below). Furthermore, we calculated the clustering coefficients from the empirical SC and FC and the characteristic path lengths from the empirical PL and FC matrices. These latter two metrics can also be used to compare our results with the literature investigating the influence of the brain parcellation on graph-theoretical metrics extracted from empirical SC and FC (Wang et al., 2009; Zalesky et al., 2010).

Finally, we sought to find correlations between the model simulation results and the extracted graph-theoretical metrics using univariate and multivariate regression approaches (Figure 1, orange). In the remainder of this section, we discuss the procedures employed at each step in detail. The source code of our analyses and connectome data have been made available elsewhere (https://jugit.fz-juelich.de/inm7/public/parcellation-modelling and https://doi.org/10.25493/81EV-ZVT; Domhof, 2021; Domhof, Jung, Eickhoff, & Popovych, 2021).

### Extraction of Empirical Connectomes

Empirical connectomes were extracted for 200 (96 males, age 28.5 ± 3.5 years) healthy, unrelated subjects from the HCP S1200 release dataset (https://www.humanconnectomeproject.org; Van Essen et al., 2012, 2013) using 19 different brain parcellations. The local ethics committee of the HCP WU-Minn gave its approval for the study and written, informed consent was given by all subjects. Here, we discuss the extraction of empirical SC and PL from dwMRI data and empirical FC from fMRI data, and present the brain atlases for which we extracted the region-based connectomes.

#### SC extraction from dwMRI.

For the extraction of the empirical SC matrices from dwMRI data, we used a workflow developed in-house that consisted of four stages: (1) preprocessing of dwMRI images, (2) calculation of the whole-brain tractography (WBT), (3) transformation of the atlas images, and (4) reconstruction of the empirical SC. The workflow included functions from the ANTs (Tustison et al., 2010), FreeSurfer (Dale, Fischl, & Sereno, 1999), FSL (Jenkinson, Beckmann, Behrens, Woolrich, & Smith, 2012), and MRtrix3 (Tournier et al., 2019) software packages. Computations were performed on the JURECA high-performance computing cluster (Jülich Supercomputing Centre, 2018).

(1) In the preprocessing stage, we used FreeSurfer functions to perform the following operations on the T1-weighted images: bias field correction, tissue segmentation, cortical (surface) reconstruction, volume-surface conversion, and surface deformation. We also used FreeSurfer functions to correct the dwMRI images with regard to head motions and eddy current distortions, while MRtrix3 functions were employed to denoise them and perform bias field correction. The dwMRI images were then registered to the T1-weighted images using the linear and nonlinear transformation functions included in FSL; afterwards, tissue segmentation was performed for these images as well. (2) Subsequently, WBT was calculated using exclusively MRtrix3 functions. A multi-shell, multi-tissue constrained algorithm (Jeurissen, Tournier, Dhollander, Connelly, & Sijbers, 2014) estimated the response functions for spherical deconvolution, which were subsequently used to determine the fiber-oriented distributions from the dwMRI data. The WBT was then completed through a second-order integration over the fiber-oriented distributions using a probabilistic algorithm (Tournier, Calamante, & Connelly, 2010), where we used 10M streamlines and the following other tracking parameter settings: step size = 0.625 mm, angle = 45°, min. length = 2.5 mm, max. length = 250 mm, FOD amplitude for terminating tract = 0.06, max. attempts per seed = 50, max. number of sampling trials = 1,000, and downsampling = 3 mm. (3) Next, the images of the brain atlases used in this study (see below) were linearly and nonlinearly transformed from the standard space (in which they were all sampled) to the native space using FSL functions. (4) Finally, we reconstructed the empirical SCs and PLs for all pairs of parcels included in a particular parcellation by using the MRtrix3 function *tck2connectome*.

#### FC extraction from fMRI.

To construct the empirical FC matrix, BOLD signals of the resting-state brain activity were first extracted from fMRI data that were preprocessed using the ICA-FIX approach as provided by the HCP repository (Griffanti et al., 2014), which eliminated the motion parameter but not the global signal effect from the images. Here, the brain atlas images were used to calculate the mean voxel intensity across each parcel per volume resulting in one BOLD signal time series per parcel. Individual time series were linearly detrended and z-scored before we constructed the empirical FC matrix by calculating the Pearson correlation coefficients across the time series for each pair of parcels. Four resting-state fMRI sessions were available in the HCP dataset for every subject (two phase encoding directions scanned on two days), each one comprising 1,200 volumes sampled with a repetition time of 720 ms. We thus calculated four different empirical FCs per subject that were used for the validation of our models.

#### Brain parcellations.

In our study, we performed the whole workflow outlined in Figure 1 for the 19 parcellations included in Table 1. As the aim of this study is to compare the modeling results for a variety of brain atlases, we ensured their comparability such that only cortical areas were considered and that all parcellations had similar volumes and were sampled to the MNI152 nonlinear template space (Grabner et al., 2006). For more details on the preprocessing of the used atlases, see the Supplementary Method.

**Table 1.** Overview of the used brain parcellation schemes with the index for reference in this study, the number of parcels after image processing, and associated publications. In addition to this table, we have included a Supplementary Data Sheet that includes (a number of statistics of) the connectomes that were extracted through the use of these parcellations.

Index	Name	No. of parcels	Refs.
1	MIST	31	Urchs et al. (2019)
2		56	
3		103	
4		167	
5	Craddock	38	Craddock, James, Holtzheimer, Hu, and Mayberg (2012)
6		56	
7		108	
8		160	
9	Shen 2013	79	Shen, Tokoglu, Papademetris, and Constable (2013)
10		156	
11	Schaefer	100	Schaefer et al. (2018)
12		200	
13	Harvard-Oxford	48	Desikan et al. (2006); Frazier et al. (2005); Goldstein et al. (2007); Makris et al. (2006)
14		96	
15	Desikan-Killiany	70	Desikan et al. (2006)
16	von Economo-Koskinas	86	Scholtens, de Reus, de Lange, Schmidt, and van den Heuvel (2018); von Economo and Koskinas (1925)
17	AAL (version 2)	92	Rolls, Joliot, and Tzourio-Mazoyer (2015); Tzourio-Mazoyer et al. (2002)
18	Destrieux	150	Destrieux, Fischl, Dale, and Halgren (2010)
19	Brainnetome	210	Fan et al. (2016)

### Graph-Theoretical Analysis of Empirical Connectomes

The empirical SC, PL, and both the empirical and the simulated FC matrices were subjected to graph-theoretical analyses in order to extract data variables portraying the properties of the networks they represent. In these analyses, the connectivity matrices represented a (network) graph in which the brain regions were the nodes and the individual matrix elements were undirected weighted edges between them. Since self-connections inferred from the empirical SC and FC extraction procedures did not influence the model simulation results (see below), we removed them from the connectivity matrices prior to the graph-theoretical analyses by setting their diagonal elements to 0. From the empirical SC and both types of FC matrices, we extracted the (weighted) degree distribution and the modularity. We selected these measures because they characterized respectively the network centrality and segregation (Rubinov & Sporns, 2010) when only the signal transmission efficiencies within the network were taken into account. The PL matrix may also provide information about the network properties from the point of view of signal transmission latencies. Here, we used the closeness centrality distribution and the global efficiency as indicators of network centrality and integration, respectively.

The degree for empirical SC and both types of FC and closeness centrality for empirical PL indicate how strongly and how quickly a node may influence the network dynamics, respectively. Accordingly, the global efficiency describes (for empirical PL) how quickly signals may be integrated throughout the network, and the modularity portrays (for empirical SC and both types of FC) to what extent the network is segregated into separate modules that have many or strong intramodular and few or weak intermodular connections.

Besides the modularity and the global efficiency, we also calculated the clustering coefficient as a measure of segregation from the empirical SC and FC and the characteristic path length as a measure of integration from the empirical PL and FC matrices. Even though the modularity and global efficiency are more state-of-the-art techniques, the calculation of the clustering coefficient and characteristic path length enabled the comparison of our study with the literature investigating the influence of the brain parcellation on the graph-theoretical measures of empirical SC and FC (Wang et al., 2009; Zalesky et al., 2010).

In the remainder of this section, we explain in detail how and why these particular metrics were calculated. Any calculations were carried out using the Python programming language (Python Software Foundation, https://www.python.org/) in combination with the SciPy (Virtanen et al., 2020), NumPy (van der Walt, Colbert, & Varoquaux, 2011), and NetworkX (Hagberg, Swart, & S Chult, 2008) modules.

#### Degree distribution.

Let a symmetric *N* × *N* coupling matrix **W** determine how the *N* network nodes are connected by undirected, weighted edges. Here, the assumption of symmetry is justified because the empirical SC and empirical and simulated FC matrices inferred from WBT and (simulated) BOLD signal time series correlations, respectively, are symmetric as well. The degree *d_j_* of node *j* can be calculated by taking the sum over the *j*th row of **W** leading to *N* values for the entire network corresponding to the number of parcels included in the used brain parcellation. We actually used the degree as opposed to other measures of centrality because of this simple summation: It makes the degree distribution easy to calculate and straightforwardly interpretable with respect to the neurobiology of the brain (Rubinov & Sporns, 2010). The degrees could be directly calculated from the empirical SC matrices. The empirical and simulated FCs were first thresholded at 0, and the Fisher Z-transforms (Fisher, 1915, 1921) of the positive elements were subsequently calculated before determining the degrees.

To compare the degree distributions across parcellations, we fitted them to the gamma (Gamma(*x*|*k*, *θ*)) parametric probability distribution. The gamma distribution is defined for positive real numbers (*x* > 0) by the following equation:

$$\text{Gamma}\left(x|k,\theta \right)=\frac{1}{\theta^{k}\Gamma \left(k\right)}x^{k-1}\exp\left(-\frac{x}{\theta }\right),$$ (1)

where Γ(*x*) represents the gamma function and *k* and *θ* are free parameters commonly referred to as the shape and scale parameter, respectively. The former determines to what extent the distribution function has a shape resembling an exponential decay or a bell curve, and the latter scales the probabilities with respect to the x-axis (see Figure S1 in the Supplementary Results for an illustration). The fitting result for SC and FC matrices comprised the fitted shape and scale parameters denoted by ${\text{Degree}}_{\text{shape}}^{SC/FC}$ and ${\text{Degree}}_{\text{scale}}^{SC/FC}$, respectively. In addition to these fitted parameters, we also calculated the Kolmogorov-Smirnov statistics between the fitted cumulative gamma distributions and the cumulative empirical degree distributions, and the mean and the standard deviation of the degree.

We used the gamma distribution to characterize the degree distribution for several reasons. First, we acknowledge that the degree can practically assume semi-infinite values because it cannot be smaller than 0 for the empirical SC as well as for the thresholded and Fisher Z-transformed empirical and simulated FC. Then, modeling the distribution by the gamma distribution is more applicable to this situation than, for example, by the Gaussian distribution. In particular, the shape parameter of the gamma distribution may reflect the variable concentrations of degrees close to 0 that are observed for the different parcellations; see the Supplementary Data Sheet. Second, studies investigating the influence of the brain parcellation on graph-theoretical measures extracted from empirical SC and FC have used the truncated power law model to characterize degree distributions (Wang et al., 2009; Zalesky et al., 2010). The truncated power law model essentially is an unnormalized version of the gamma distribution (see Wang et al., 2009; Zalesky et al., 2010; and Equation 1). Therefore, the parameters of the gamma distribution and the truncated power law model are practically the same. Using the gamma distribution to characterize the degree distribution thus enhances the comparability of our study with the literature. Nevertheless, we deviate from the use of the (unnormalized) truncated power law model as the normalization condition enables the comparison of the fitting errors between the empirical and fitted distributions across parcellations. The latter is our third and final reason to use the gamma distribution to model the degree distributions. In sum, the gamma distribution suits the problem at hand given that the degrees can only assume values larger than or equal to 0. In addition, it enables the comparison of all the fitting results across parcellations, and enhances the comparability of our results with the literature.

#### Modularity.

The modularity of a network was obtained by maximizing its expression (Rubinov & Sporns, 2011):

$$\text{Modularity}=\frac{1}{w^{+}}\sum_{i=1}^{N}\sum_{j=1}^{N}\left(W_{ij}^{+}-e_{ij}^{+}\right)\delta_{M_{i},M_{j}}-\frac{1}{w^{+}+w^{-}}\sum_{i=1}^{N}\sum_{j=1}^{N}\left(W_{ij}^{-}-e_{ij}^{-}\right)\delta_{M_{i},M_{j}}.$$ (2)

Here *i* and *j* both represent the number of a particular network node. Additionally, $W_{ij}^{+}$ and $W_{ij}^{-}$ are the positive and negative elements of **W**, respectively (i.e., if *W_ij_* > 0, then $W_{ij}^{+}$ = *W_ij_* and $W_{ij}^{-}$ = 0; otherwise $W_{ij}^{+}$ = 0 and $W_{ij}^{-}$ = −*W_ij_*). Then *w*^±^ represents the total sum over $W_{ij}^{\pm }$, and $e_{ij}^{\pm }$ is defined by

$$e_{ij}^{\pm }=\frac{\sum_{j=1}^{N}W_{ij}^{\pm }\sum_{i=1}^{N}W_{ij}^{\pm }}{w^{\pm }}.$$ (3)

Finally, *M_i_* denotes the module to which node *i* belongs and $\delta_{M_{i},M_{j}}$ is the Kronecker delta, meaning $\delta_{M_{i},M_{j}}$ = 1 if *M_i_* = *M_j_* and $\delta_{M_{i},M_{j}}$ = 1 otherwise. By changing the modular structure of the network (i.e., changing *M_i_*), the modularity can be maximized. Since evaluating all possible module configurations is too computationally expensive, we used the Louvain algorithm to solve this optimization problem (Blondel, Guillaume, Lambiotte, & Lefebvre, 2008).

The modularity was selected from other measures of segregation (e.g., the clustering coefficient and local efficiency) because of its more sophisticated design, especially in light of the negative correlations an FC matrix can have (Rubinov & Sporns, 2010, 2011). Additionally, it allows for an in-depth examination of the modular network structure after the maximization has been performed, for instance, to determine the strength of community structure for a given network (Newman & Girvan, 2004).

#### Closeness centrality.

Signals propagating throughout the network from one node to another can traverse several edges that have associated weights representing the cost of crossing them. The minimal cost of traveling between nodes *i* and *j* is termed the shortest path length *l_ij_*. For the empirical PL matrix, the calculated shortest path length literally estimated the minimal distance that the signals have to cover along the white matter fibers connecting two brain regions. The closeness centrality *L_j_* of node *j* is then defined as the inverse of the mean shortest path length between that node and all other nodes in the network (Rubinov & Sporns, 2010):

$$L_{j}=\frac{N-1}{\sum_{i=1}^{N}l_{ij}},\;\text{where}\;l_{ii}=0.$$ (4)

We calculated the closeness centrality for all nodes to determine the network’s closeness centrality distribution. Subsequently, we fitted this distribution to the gamma probability distribution (1) because also the closeness centrality could not assume values below 0, which resulted in the fitted gamma distribution shape and scale parameters denoted by $\text{Centr}._{\text{shape}}^{PL}$ and $\text{Centr}._{\text{scale}}^{PL}$, respectively. Just as with the degree distribution, we also calculated the root-mean-square errors between the fitted cumulative gamma distributions and the cumulative empirical closeness centrality distributions, and the mean and the standard deviation of the closeness centrality.

Also the degree or betweenness centrality could have been used to analyze the empirical PL matrix (Rubinov & Sporns, 2010). Nevertheless, we selected the closeness centrality as opposed to these alternatives. The degree calculated on the basis of the empirical PL does not have the same neurobiological interpretation as with the empirical SC and both types of FC (see above). The betweenness centrality has the disadvantage that it discards any information about the shortest path lengths themselves (Rubinov & Sporns, 2010).

#### Global efficiency.

The global efficiency of a network was also defined in terms of the shortest path lengths (Rubinov & Sporns, 2010):

$$\text{Efficiency}=\frac{1}{N}\sum_{i=1}^{N}\frac{\sum_{j=1}^{N}l_{ij}^{-1}}{N-1},\;\text{where}\;l_{ii}=0.$$ (5)

It can thus be interpreted as the mean of the inverted shortest path lengths across all pairs of network nodes. An alternative measure of integration is the characteristic path length (Rubinov & Sporns, 2010), but it has been argued that global efficiency may be superior when investigating brain networks (Achard & Bullmore, 2007).

#### Clustering coefficient.

For weighted graphs, which we consider in our study, several variants of the clustering coefficient exist. We use the expression of the clustering coefficient proposed by Onnela, Saramäki, Kertész, and Kaski (2005):

$$\text{Cluster}=\frac{1}{N}\sum_{i=1}^{N}\frac{\sum_{j=1}^{N}\sum_{k=1}^{N}{\left({\hat{W}}_{ij}{\hat{W}}_{ik}{\hat{W}}_{jk}\right)}^{1/3}}{d_{i}\left(d_{i}-1\right)}.$$ (6)

Here, $\hat{W}$*_ij_* = *W_ij_*/max(**W**) are the elements of the connectivity matrix normalized by their maximum and *d_i_* represents the degree of node *i*.

The clustering coefficient is a rather simple measure of segregation and its expression has not been optimized for FC matrices. Therefore, we consider the modularity to be a more accurate statistic for network segregation. Nevertheless, as previous work studying the influence of brain parcellations on graph-theoretical measures extracted from empirical connectomes included this measure (Wang et al., 2009; Zalesky et al., 2010), we have added it to our calculations. We calculated the clustering coefficient from the empirical SC matrix and from the thresholded and Fisher’s Z-transformed empirical FC matrix (see also the case with the degree).

#### Characteristic path length.

The characteristic path length is obtained by averaging the shortest path length across all pairs of nodes (Rubinov & Sporns, 2010):

$$\text{Char}.\;PL=\frac{1}{N}\sum_{i=1}^{N}\frac{\sum_{j=1}^{N}l_{ij}}{N-1},\;\text{where}\;l_{ii}=0.$$ (7)

Analogous to the modularity and the clustering coefficient describing the network segregation, the global efficiency and the characteristic path length are both measures of network integration. As mentioned above, the global efficiency is superior in brain network research (Achard & Bullmore, 2007). However, we also included the characteristic path length to ameliorate the comparability of our work with other studies investigating the influence of the brain parcellation on region-based SC and FC by means of this metric (Wang et al., 2009; Zalesky et al., 2010). We calculated the characteristic path length associated with the structural connectivity by using the PL matrix. For the functional connectivity we used the thresholded and Fisher Z-transformed empirical FC matrix with inverted elements. The latter inversion was done after the Z-transformation to convert the functional association strengths to estimations of the link lengths, where link strengths and lengths are inversely related (Rubinov & Sporns, 2010).

### Model Simulations

In the modeling stage of our workflow, the brain was once again seen as a network of brain regions (network nodes) parcellated according to a given brain atlas. We subsequently used a system of coupled oscillators to model the collective dynamics of the mean-field activities of the individual brain regions. The coupling between network nodes was defined by the extracted empirical SC, where the SC matrix determined how strongly one region influenced the other. The PL matrix was used to evaluate the latency in the signal propagation between the nodes. By simulating the dynamics of the whole-brain models, we sampled the activity time series of the *N* nodes included in the network. We subsequently correlated these time series with one another and constructed a simulated FC matrix. Finally, the similarity between the simulated and the empirical FC matrices was quantified by vectorizing the upper triangular parts of both matrices excluding the diagonal and subsequently calculating the Pearson correlation coefficient between the resulting two vectors. By exploring the parameter space of the model via a grid search, the maximal similarity between the empirical and simulated FC matrices could be found, which is henceforth also referred to as the *goodness-of-fit* of the model.

In this study, we modeled the local dynamics of the brain regions from different perspectives by considering two different models. The first model was the Kuramoto system of coupled phase oscillators (Kuramoto, 1984), and the other was an ensemble of Wilson-Cowan type neural mass models (Wilson & Cowan, 1972). These two models were chosen because of their major conceptual differences, which increased the likelihood of finding cross-model deviations. These models have also been used in previous studies investigating the brain’s structure-function relationship by dynamical whole-brain models (Deco, Jirsa, McIntosh, Sporns, & Kötter, 2009; Messé, Rudrauf, Benali, & Marrelec, 2014; Ponce-Alvarez et al., 2015).

#### Phase oscillator model.

In the Kuramoto model (Kuramoto, 1984), the mean-field activity of brain region *i* ∈ {1, 2, …, *N*} (*N* is the number of brain regions in a given parcellation) is assumed to oscillate with a region-specific frequency *f_i_*, and the dynamics of its phase *φ_i_*(*t*) are governed by the differential equation

$${\dot{\varphi }}_{i}\left(t\right)=2\pi f_{i}+\sum_{j=1}^{N}C_{ij}\sin\left(\varphi_{j}\left(t-\tau_{ij}\right)-\varphi_{i}\left(t\right)\right)+\sigma_{p}\nu_{i}\left(t\right).$$ (8)

Here *ν_i_*(*t*) is independent Gaussian white noise with zero mean and unit variance, and σ_p_ = 0.17 is the noise intensity. Furthermore, *C_ij_* and *τ_ij_* represent the individual coupling strength and delay values between brain regions, respectively. These were derived from the empirical SC and PL matrices via

$$C_{ij}=\left\{\begin{array}{ll} 0 & \text{if}\;i=j\\ G.\frac{{SC}_{ij}}{N\langle SC\rangle } & \text{otherwise} \end{array}\right.\;\text{and}\;\tau_{ij}=\left\{\begin{array}{ll} 0 & \text{if}\;i=j\\ \tau .\frac{{PL}_{ij}}{\langle PL\rangle } & \text{otherwise} \end{array}\right..$$ (9)

Here, the operator 〈·〉 returns the mean over all elements of the matrix, and *G* and *τ* are scaling factors referred to as the global coupling and global delay.

Although the Kuramoto model has been used in different paradigms in relation to large-scale whole-brain models (e.g., Messé et al., 2014 vs. Ponce-Alvarez et al., 2015), we adopted the approach wherein the ultraslow phase dynamics of the BOLD signals was directly modeled by *φ_i_*(*t*). Then the simulated BOLD signals cos(*φ_i_*(*t*)) were used for the calculation of the simulated FC matrix. The region-specific oscillation frequencies *f_i_* in the range [0.01, 0.1] Hz were derived from the empirical BOLD signal time series via spectral density estimation. For this analysis, we subjected those signals to Welch’s method (*welch* function implemented in the SciPy module; Virtanen et al., 2020) while using a 1,024 time-points-long Hamming window function with 95% (927 time points) overlap between segments. We used the frequencies corresponding to the largest peaks in the spectra and heterogenized them a little by adding Gaussian white noise with zero mean and 0.002 Hz standard deviation. Finally, *G* and *τ* were considered to be free parameters and were optimized in order to maximize the similarity between empirical and simulated FC.

#### Neural mass model.

The neural mass model used in this study was a Wilson-Cowan model (Wilson & Cowan, 1972) adapted from the paper by Deco et al. (2009). It models the interaction between the excitatory and inhibitory neuron ensembles of the *i*th brain region, where their mean firing rates *E_i_*(*t*) and *I_i_*(*t*), that is, the proportion of cells firing within a unit of time, respectively, are modeled via the following coupled differential equations:

$$\mu_{E}{\dot{E}}_{i}\left(t\right)=-E_{i}\left(t\right)+\kappa S\left(\sum_{j=1}^{N}C_{ij}E_{j}\left(t-\tau_{ij}\right)-c_{EI}I_{i}\left(t\right)+I_{b}\right)+\sigma_{n}\nu_{i}\left(t\right)\;\text{and}$$ (10)

$$\mu_{E}{\dot{I}}_{i}\left(t\right)=-I_{i}\left(t\right)+\kappa S\left(c_{IE}E_{i}\left(t\right)\right)+\sigma_{n}\nu_{i}\left(t\right).$$ (11)

In these equations, *μ_E_* and *μ_I_* are the decay time constants of the excitatory and inhibitory activity, respectively. Both populations received the same zero-mean, independent Gaussian white noise of intensity *σ_n_*. Parameters *c_EI_* and *c_IE_* regulate the inhibition of the excitatory cells by the inhibitory pool and the excitation of the inhibitory cells by the excitatory pool, respectively. S(*x*) is a sigmoid function defined by

$$S\left(x\right)=\frac{1}{1+\exp\left(-\lambda \left(x-\gamma \right)\right)}-\frac{1}{1+\exp\left(\lambda \gamma \right)},$$ (12)

where *λ* and *γ* determine its width and the position of its inflexion point, respectively. Additionally, *I_b_* is a constant external input to the excitatory neurons, and *κ* = (1 + exp(*λγ*))/exp(*λγ*) scales S(*x*) such that *κ*S(*x*) = 1 as *x* → ∞. Finally, *C_ij_* and *τ_ij_* have the same interpretations and similar associated expressions as with the Kuramoto model (9):

$$C_{ij}=\left\{\begin{array}{ll} c_{EE} & \text{if}\;i=j\\ G.\frac{{SC}_{ij}}{N\langle SC\rangle } & \text{otherwise} \end{array}\right.\;\text{and}\;\tau_{ij}=\left\{\begin{array}{ll} 0 & \text{if}\;i=j\\ \tau .\frac{{PL}_{ij}}{\langle PL\rangle } & \text{otherwise} \end{array}\right.,$$ (13)

where *c_EE_* is a parameter scaling the self-excitation of the excitatory pool.

We set the model parameters to the values listed in Table 2. As for the Kuramoto model, parameters *G* and *τ* were regarded as free parameters and were varied to maximize the similarity between the empirical and simulated FC matrix. The considered parameter configurations resulted in a low activity state of disconnected nodes (*G* = 0) and generation of limit-cycle oscillations with an alpha-band frequency when the individual regions were coupled (*G* > 0). The modeled alpha oscillations have been shown to be dominant in EEG of human resting-state brain activity (Fraga González et al., 2018; Spitoni, Di Russo, Cimmino, Bozzacchi, & Pizzamiglio, 2013) and to interact with BOLD responses (Mayhew, Ostwald, Porcaro, & Bagshaw, 2013).

**Table 2.** Parameter settings of the neural mass model

**Parameter**	**Value**	**Parameter**	**Value**	**Parameter**	**Value**
*μ_E_*	20 ms	*λ*	20.000	*c_EE_*	1.000
*μ_I_*	20 ms	*γ*	0.300	*c_EI_*	1.500
*I_b_*	0.100 ms	*σ_n_*	0.002	*c_IE_*	0.000

Simulating the neural mass model yielded neuronal signal time series that are not directly comparable with the empirical BOLD responses extracted from fMRI data. To account for this, the neuronal signals of the excitatory pool were converted to BOLD responses by the Balloon-Windkessel model from Friston, Harrison, and Penny (2003), a procedure that has also been used elsewhere (Havlicek et al., 2015). The resulting (simulated) BOLD signals were subsequently used to construct the simulated FC matrix.

#### Implementation, simulation, and parameter variation.

The Python (Python Software Foundation, https://www.python.org/) and C++ (Standard C++ Foundation, https://isocpp.org/) programming languages were selected for the implementation of the model simulations; here, we also used the SciPy (Virtanen et al., 2020) and Numpy (van der Walt et al., 2011) modules. Simulation and analysis computations were carried out on the JURECA high-performance computing cluster (Jülich Supercomputing Centre, 2018). The temporal integration of both models as well as the neuronal to BOLD signal conversion followed Heun’s method. For both models, we optimized the free parameters by simulating the models using a dense grid of 64 × 48 parameter points for the global coupling and delay, respectively, and subsequently selecting the parameters maximizing the correlation between the empirical and simulated FC (goodness-of-fit). Regarding the phase oscillator model, the global coupling and delay were varied using *G* ∈ {0.000, 0.015, 0.030, …, 0.945} and *τ* ∈ {0 s, 1 s, 2 s, …, 47 s}. For every parameter setting, we then simulated 70 min of network dynamics with a 60-ms integration time step and disregarded the first 10 min so that the initial conditions did not influence the results. For the neural mass model we used *G* ∈ {0.000, 0.018, 0.036, …, 1.134} and *τ* ∈ {0.0 ms, 1.5 ms, 3.0 ms, …, 70.5 ms} for the global coupling and delay, sampled 510 s of network activity with an integration step size of 2 ms and removed the first 150 s prior to analysis. The differences in the simulation parameters (simulated time and integration time step size) between both models were adapted to the ultraslow timescale and alpha frequency oscillations of the phase oscillators and the neural mass model, respectively. The simulations above were performed individually for each combination of the 200 subjects, the 2 models, and the 19 considered parcellations listed in Table 1.

### Analysis

#### Analysis of interparcellation variations.

We observed differences across brain parcellations when examining the graph-theoretical measures and goodness-of-fit. We determined whether these deviations were consistent across subjects; in other words, we assessed whether altering the parcellation changes the patterns of the values across all subjects. To this end, we gathered the values of the considered graph-theoretical measure for the individual subjects into separate data vectors for each parcellation and calculated the Pearson correlation coefficient corresponding to each pair of vectors and thus parcellations. The same approach was used to investigate goodness-of-fit correlations across subjects for different models, where separate data vectors were constructed for every combination of brain atlas and model for local dynamics to also assess the effect of the model in this respect.

Then, we studied whether covariations between the graph-theoretical metrics and the goodness-of-fit existed by combining principal component analysis with ordinary least squares regression. We built a dataset with the granularities (number of parcels *N*), the median values across subjects of 13 considered graph-theoretical measures extracted from the empirical SC, PL, and FC matrices, and the Pearson correlation coefficient between the empirical SC and empirical FC such that we obtained a 15 × 19 matrix in which each row was associated with one of those statistics and each column held the values of those metrics for a particular parcellation from Table 1. The dataset was z-scored to ensure the comparability of the individual metrics to one another and subsequently decomposed into the scores and loadings corresponding to the principal components (PCs) through the use of singular value decomposition as performed by the *linalg.svd* function in NumPy (van der Walt et al., 2011). Finally, the scores of the PCs were regressed with the median values of the goodness-of-fit across subjects for every brain atlas for both model types separately. Here, we considered both a univariate and a multivariate approach, in which we used the scores of only the first PC and those of multiple PCs, respectively, to explain the variance in the goodness-of-fit for varying brain parcellation via ordinary least squares regression.

#### Detection of within-parcellation, between-subject correlations.

We checked whether the covariations found between the group-averaged graph-theoretical measures and the goodness-of-fit across parcellations were also present when considering intraparcellation, interindividual variations. Hence, we investigated whether graph-theoretical metrics could also explain interindividual differences when considering a specific parcellation in isolation. First, we wielded the same approach from the previous paragraph for this investigation. For each brain atlas, we built a 14 × 800 data matrix, in which each row corresponded to one of the data variables mentioned in the previous paragraph excluding the granularity and each column held the values of these statistics for a specific subject and fMRI session pair. For the HCP dataset used in our study, four resting-state fMRI sessions were available for each subject, which led to the 200 (subjects) × 4 (fMRI sessions) = 800 columns in the datasets. In order to keep the matrix dimensions the same also for the SC matrices, the same SC characteristics were repeated in the dataset for the individual fMRI sessions per subject. We calculated the z-scored dataset, extracted the first PC, and regressed its scores with the goodness-of-fits of the individual subjects.

We also checked whether a multivariate approach could substantially improve the explained interindividual variance in the goodness-of-fit across subjects for a given brain parcellation. To do so, we directly regressed the z-scored dataset with the goodness-of-fits of the individual subjects and sessions via (multivariate) ordinary least squares regression for the two models separately.

## RESULTS

In this study, we investigated the effect of the brain atlas on the goodness-of-fit of dynamical whole-brain models. For this inquiry, we first extracted the empirical SC, PL, and FC matrices from the dwMRI and fMRI data of 200 subjects included in the HCP S1200 release dataset using the 19 parcellations in Table 1 and subsequently subjected them to graph-theoretical analyses. Next, we sampled the modeling results associated with those empirical SC and FC matrices for the Kuramoto system (8–9) of coupled phase oscillators (Kuramoto, 1984) and the ensemble (10–13) of Wilson-Cowan type neural mass models (Wilson & Cowan, 1972). Finally, we investigated through principal component analysis and linear regressions whether differences in network properties could explain the variance in modeling results.

### Parcellation-Induced Heterogeneity of Empirical Connectomes

**Figure 2.**
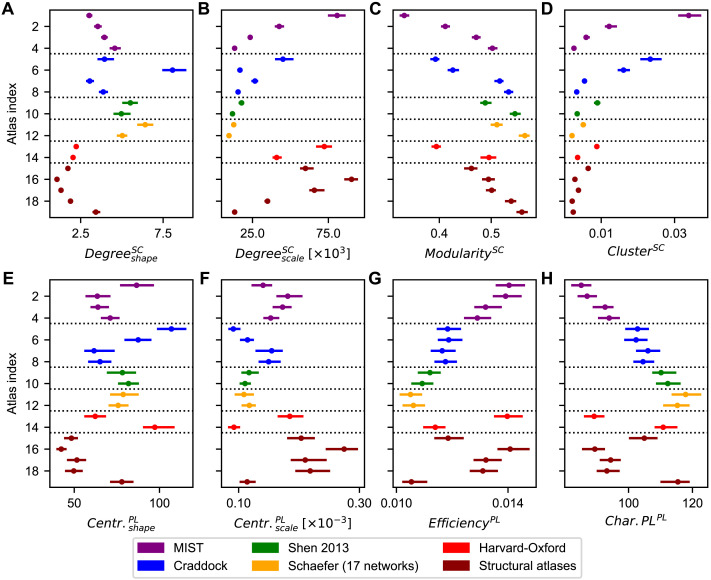
Heterogeneity of graph-theoretical properties of empirical structural networks across parcellations. (**A**–**D**) Statistics extracted from the structural connectivity (SC) matrices, which are the shape (A) and scale (B) parameters of the degree distributions, the modularities (C), and the clustering coefficients (D). (**E**–**H**) Statistics extracted from the path length (PL) matrices, which are the shape (E) and scale (F) parameters of the closeness centrality distributions, the global efficiencies (G), and the characteristic path lengths (H). Dots and lines depict the medians and interquartile ranges across subjects, respectively. The atlas indices on the vertical axes correspond to those in Table 1, which contains the information about the used parcellations. Abbreviations: Centr. = closeness centrality, Char.PL = characteristic path length.

We found a high variability in the graph-theoretical network properties of the empirical SC for varying parcellations (Figure 2). Note, however, that the shape and scale parameters of the degree distributions of the empirical SC should be considered with some reservation as they may not fully capture all differences in these distributions across parcellations; see Supplementary Data Sheet. Nevertheless, we on average obtained better fit with the gamma distribution for all approximated network metrics than with the Gaussian distribution.

The shape parameter of the degree distribution of the empirical SC, for instance, had a median value ranging from 1.1 for the von Economo-Koskinas atlas (atlas index 16) to 8.1 for the Craddock parcellation with 56 parcels (atlas index 6) (Figure 2A). Its scale parameters exhibited an opposing trend with respect to the variation of the parcellation when compared with the shape parameters: Relatively large values for the shape parameter were accompanied by relatively small values for the scale parameter when considering an individual atlas (Figure 2B). This opposing trend was also observed for the shape parameter and scale parameters describing the closeness centrality distribution of the empirical PL matrix (Figure 2E–F). The modularities derived from the empirical SC matrix showed an increasing trend when the number of parcels grew (Figure 2C). On the other hand, the clustering coefficients showed an opposing trend (Figure 2D). This is a rather striking observation, because both measures reflect network segregation. However, the modularity is calculated through a consideration of the entire network (Equation 2); whereas the clustering coefficient is determined on a node-by-node basis (Equation 6). These findings therefore demonstrate that parcellations with higher granularities may yield structural networks that contain more pronounced subnetworks, but have fewer triplets of nodes that are strongly interconnected. The decreasing trend of the (raw) clustering coefficient with increasing granularity was also observed in other studies investigating the empirical SC (Zalesky et al., 2010). Simple dependencies on the granularity were found neither for the parameters of the degree distribution (Figure 2A–B) nor for the graph-theoretical metrics derived from the empirical PL matrix (Figure 2E–H).

Analogous to the modularity and the clustering coefficient, the global efficiency and characteristic path length of the PL matrix also exhibited opposing trends (Figure 2G–H). These opposing trends were expected: Longer characteristic path lengths reflect slower integration of signals throughout the network, which agrees with a lower global efficiency. In addition to the fitted gamma distribution parameters of the degree and closeness centrality distributions shown in Figure 2A–B and Figure 2E–F, respectively, we also calculated the means and standard deviations of the degrees and closeness centralities and the Kolmogorov-Smirnov statistics characterizing the qualities of the gamma distribution fittings; these are included in the Supplementary Results (Figure S2A–F).

**Figure 3.**
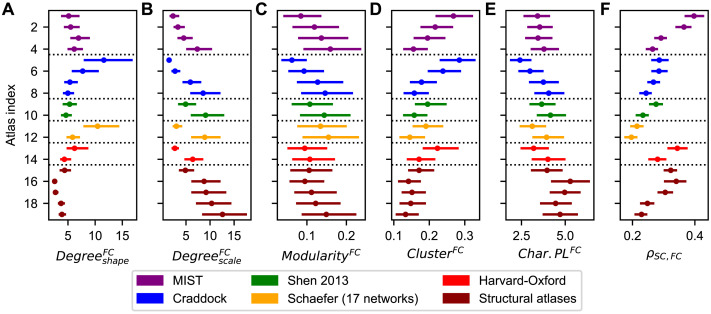
Heterogeneity of graph-theoretical properties of the empirical functional connectivity (FC) across parcellations. (**A**–**E**) Statistics extracted from the empirical FC matrices, which are the shape (A) and scale (B) parameters of their degree distributions, their modularities (C), their clustering coefficients (D), and their characteristic path lengths (E). (**F**) Pearson correlation coefficients corresponding to the structure-function relationship between the upper triangular parts (excluding diagonal) of the empirical SC and FC matrices. Dots and lines depict the medians and interquartile ranges across subjects, respectively. The atlas indices on the vertical axes correspond to those in Table 1, which contains the information about the used parcellations. Abbreviations: Char.PL = characteristic path length.

The shape parameter of the degree distribution of the empirical FC matrix exhibited similar variations across parcellations when compared with its structural counterpart (Figure 2A vs. Figure 3A), though using the Craddock parcellation with 38 parcels (atlas index 5) and the Schaefer parcellation with 100 parcels (atlas index 11) did result in some notably larger values for this statistic (Figure 3A). The scale parameter, on the other hand, seemed to mostly depend on the granularity (number of brain regions) of the parcellations (Figure 3B). Just as with the SC matrix, the modularity and the clustering coefficient of the FC matrix exhibited opposing trends, and again appeared to mostly depend on the granularity (Figure 3C–D). The characteristic path length calculated from the empirical FC did not exhibit such a general trend (Figure 3E). We also calculated the strength of the structure-function relationship as given by the Pearson correlation coefficient between the empirical SC and FC matrices (*ρ*_*SC*,*FC*_). It seemed to demonstrate similarities with the scale parameters of the degree distributions of the empirical SC and the scale parameters of the closeness centrality distributions and the global efficiencies of the PL matrix as the parcellation varies (Figure 2B, E, F, Figure 3D).

**Figure 4.**
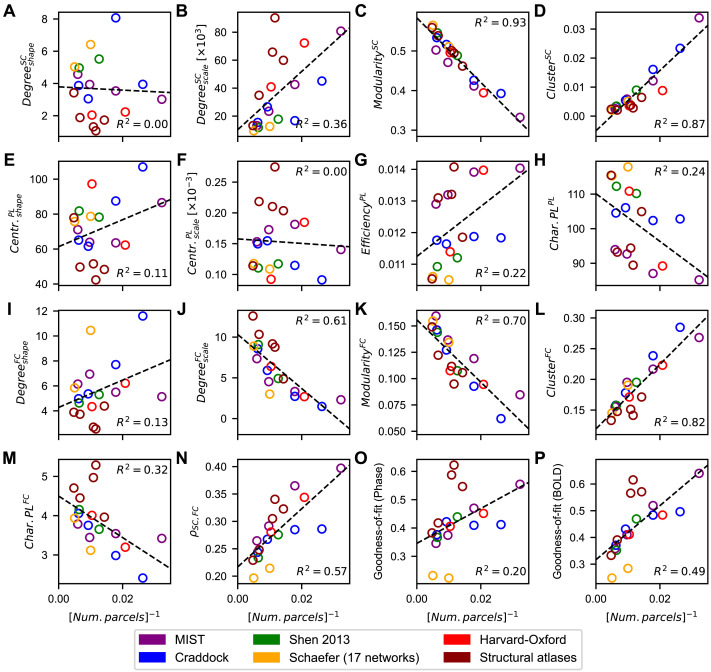
Scatterplots of all the measures shown in Figure 2 (A–H), Figure 3 (I–N), and Figure 6A (O–P) as a function of the inverse of the number of parcels included in the considered parcellations. Each dot corresponds to a particular atlas and the dashed lines show the least squares linear regressions between these points. The coefficients of determination are also displayed in each plot. Abbreviations: Centr. = closeness centrality, Char.PL = characteristic path length, Num. = number of.

So far, we observed trends for some graph-theoretical statistics that exhibited large dependencies on the parcellation granularity. We therefore investigated this effect in more detail. The literature shows that (graph-theoretical) statistics extracted from empirical SC and FC may be inversely related to the number of parcels included in a parcellation (Messé, 2020; Zalesky et al., 2010). We therefore plotted the median of each considered measure as a function of the inverted number of parcels for each parcellation, which revealed high dependencies on the granularity for some statistics (Figure 4A–N). Indeed, the modularity and clustering coefficient reflecting the segregation of the empirical SC and FC are highly influenced by the parcellation granularity (Figure 4C, D, K, L). The structure-function relationship *ρ*_*SC*,*FC*_ is also governed by the number of regions to a large extent (Figure 4N), which is in agreement with the results of Messé (2020). However, most of the other network properties only weakly to moderately correlate with parcellation granularity. In addition to the inverted relationship, we checked whether the granularity effect could be modeled better by a linear dependence on the number of parcels. The opposite was true: A linear treatment of the granularity effect did not lead to higher explained variances, and for many measures it even resulted in lower coefficients of determination.

To investigate how the considered measures depend on the parcellations beyond the granularity effect, we regressed this effect out by fitting the data to an inverse relationship (*y* = *a*/*N* + *b*) and examined the residuals. As expected, the residuals of the modularities and clustering coefficients exhibited differences between brain atlases that had a lower scale than the raw data; see for example Figure 4C, D, K, I vs. Figure S5C, D, K, I in the Supplementary Results. The other residuals still exhibited differences across parcellations of the same magnitude; see Figure S3, Figure S4, and Figure S5A–N in the Supplementary Results. In sum, even though the granularity of a parcellation can greatly influence some of the network statistics extracted from the empirical data, the observed parcellation-induced deviations typically go beyond such a simple relationship. We further analyze this dependence below (Interparcellation Variations of Empirical Connectomes and Modeling Results section).

**Figure 5.**
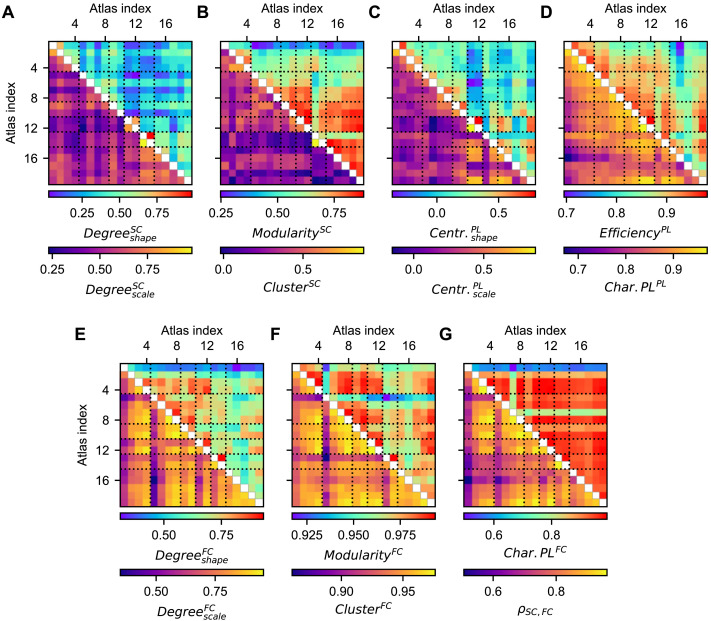
Cross-correlations across subjects of the network statistics derived from the empirical structural and functional connectomes for different parcellations. The correlations between parcellations were calculated for (**A**) the shape (upper triangle) and scale (lower triangle) parameters of the degree distributions of the empirical SC matrix, (**B**) the modularities (upper triangle) and clustering coefficients (lower triangle) of the empirical SC, (**C**) the shape (upper triangle) and scale (lower triangle) parameters of the closeness centrality distributions of the empirical PL matrix, (**D**) the global efficiencies (upper triangle) and characteristic path lengths (lower triangle) of the empirical PL matrix, (**E**) the shape (upper triangle) and scale (lower triangle) parameters of the degree distribution of the empirical FC matrix, (**F**) the modularities (upper triangle) and clustering coefficients (lower triangle) of the empirical FC, and (**G**) the characteristic path lengths of the empirical FC (upper triangle) and the Pearson correlation between the empirical SC and FC (lower triangle). The atlas indices correspond to those in Table 1, which contains the information about the used parcellations. Abbreviations: Centr. = closeness centrality, Char.PL = characteristic path length.

Subsequently, we investigated how the graph-theoretical network properties of the individual subjects correlated between each pair of the considered brain atlases; see Materials and Methods (*Analysis*) for details of this analysis. Following this procedure, we evaluated whether the interindividual differences in the empirical network statistics exhibited similar patterns between the parcellations used for the extraction of the empirical connectomes. We found that these correlations were highest for the global efficiency and characteristic path length of the empirical PL matrix (Figure 5D), for the modularity, clustering coefficient, and characteristic path length of the empirical FC matrices (Figure 5F, G), and for the correlation between empirical SC and FC (Figure 5G). Such correspondences were generally lower for the parameters of the degree and closeness centrality distributions (Figure 5A, C, E), and the modularity and clustering coefficient of the empirical SC (Figure 5B). These network metrics of the corresponding connectivity matrices are thus sensitive to a selected brain parcellation. At the individual level, network segregation properties of the empirical FC and network integration statistics thus seemed to be influenced much less by the brain parcellation than measures reflecting the centrality and the network segregation of empirical SC.

### Parcellation-Induced Heterogeneity of Modeling Results

**Figure 6.**
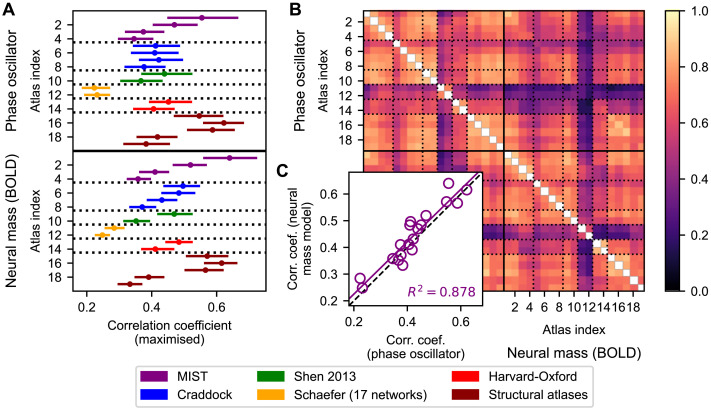
Goodness-of-fit of the whole-brain models based on coupled phase oscillators and neuronal mass models and their interrelations for the considered parcellation schemes. (**A**) Maximized correlations (goodness-of-fit) between the empirical and simulated FC matrices for the brain parcellation schemes and models investigated in this study as indicated on the vertical axes. Dots and lines depict the medians and interquartile ranges across subjects, respectively. (**B**) Correlations across subjects of the goodness-of-fit of the model between the considered parcellations and models. Table 1 contains the parcellation information corresponding to the atlas indices used in the plots. (**C**) Scatterplot of the medians of the goodness-of-fit corresponding to the phase oscillator (x-axis) and neural mass model (y-axis) across subjects. Each dot corresponds to a particular parcellation, the purple line portrays the linear regression between both types of goodness-of-fit, and the black dashed line corresponds to *x* = *y*.

In this section we present the results of the model simulations for all brain atlases in Table 1 and the two considered whole-brain models of coupled phase oscillators (8–9) and neural mass models (10–13). For each combination of subject, parcellation, and model, the optimal values of the global coupling and delay parameters were found by maximizing the Pearson correlation between the empirical and simulated FC matrices, which provided the goodness-of-fit of the model illustrated in Figure 6A for both models. For varying parcellations we observed a high variability of the fitting results, implying that the extent of correspondence between simulated and empirical FC strongly depended on the selected parcellation. Here, the MIST parcellation with 31 parcels, the Desikan-Killiany atlas, the von Economo-Koskinas atlas, and the AAL atlas yielded the highest goodness-of-fits independently of the model type (Figure 6A, atlas indices 1, 16, 17, and 18, respectively). Interestingly, the interindividual variance of the goodness-of-fit had approximately the same range as the structure-function relationship between the empirical SC and FC matrices (Figure 3). It also appeared as if the patterns of the goodness-of-fit versus parcellations were similar to each other for different models (Figure 6A).

To quantify the mentioned similarity, we considered the medians of the goodness-of-fit calculated over all subjects corresponding to the phase oscillators and regressed them across parcellations with those of the neural mass model. This resulted in a regression with a coefficient of determination of 0.88 (Figure 6C), suggesting a model-independent impact of a given brain parcellation on the (group-averaged) goodness-of-fit. As with the graph-theoretical measures, we investigated the effect of granularity on the goodness-of-fit by plotting its median across subjects against the inverse of the number of parcels included in the parcellations. The corresponding plots exhibited moderate correlations (Figure 4O–P), where the impact of granularity on the fitting results for the phase model is much smaller than that for the neural mass model. To quantify the parcellation-induced influence on the goodness-of-fit beyond the dependence on the granularity, the effect of the (inverted) granularity was regressed out. The residual goodness-of-fits exhibited variations across parcellations that had similar magnitudes as the original data; see for example Figure 4O–P versus Figure S5O–P in the Supplementary Results. In addition, the agreement between models was further enhanced; see Figure 6C versus Figure S6C. In conclusion, the granularity influences the goodness-of-fit to a limited extent, implying that the parcellation-induced deviations cannot exclusively be explained by this quantity.

The goodness-of-fit was also correlated across individual subjects between the considered parcellations and models to evaluate how similar the patterns of the model fitting over all subjects were for different parcellations and models; see Materials and Methods (*Analysis*) for details of this analysis. The results showed relatively high correspondence of the fitting patterns across individual subjects for many of the parcellation combinations for the same as well as for different models, which is illustrated in Figure 6B. Nevertheless, we also observed generally lower correlations for the Schaefer and also the Harvard-Oxford atlases, both within and across models (Figure 6B, atlas indices 11–14). Note that we did not find such clear, generally decreased values when considering the correlations of the empirical graph-theoretical statistics across parcellations (Figure 5). For the empirical FC matrices, the Craddock atlas with 38 parcels could however be distinguished in this respect (Figure 5E–F, atlas index 5), and only a slight indication of a lower correlation could be found for the scale parameter of the degree distribution of the empirical FC for the Schaefer atlas with 100 parcels and Harvard-Oxford atlas with 48 parcels (Figure 5E, atlas indices 11 and 13).

Taken together, the modeling results as represented by the goodness-of-fit between empirical and simulated FC showed pronounced heterogeneity with respect to the variation of the brain atlas. Additionally, we found that the intersubject variability of the fitting results exhibited similar patterns for most of the considered parcellations, although we also observed some exceptions for which this correspondence is limited (the Schaefer and Harvard-Oxford atlases).

### Interparcellation Variations of Empirical Connectomes and Modeling Results

**Figure 7.**
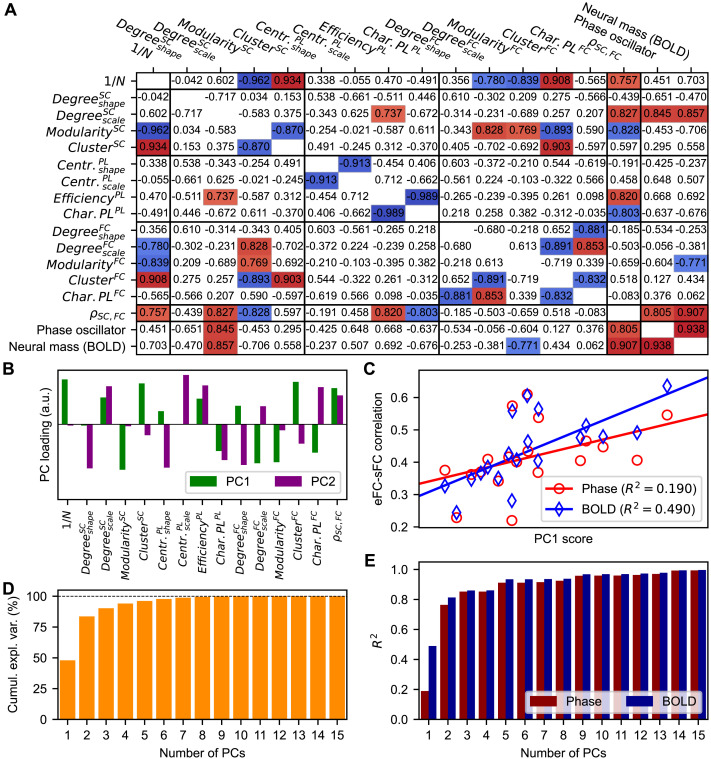
Relationship between the interparcellation variations of the empirical graph-theoretical metrics and the goodness-of-fit. (**A**) Cross-correlations among the inverted granularities, the graph-theoretical measures of the empirical connectomes (network propertties depicted in Figure 2 and Figure 3), the structure-function relationship, and the goodness-of-fit of the models to the empirical data. The corelation was calculated across parcellations between the median values over all subjects. Significant correlations are highlighted by colors (*p* < 0.05, two-sided, Bonferroni corrected). (**B**) Loadings of the first (PC1) and the second (PC2) principal components of the group-averaged graph-theoretical metrics, that is, the contributions of the original empirical data variables to PC1 and PC2. (**C**) Regressions of the PC1 scores with the medians of the goodness-of-fit between empirical (eFC) and simulated (sFC) functional connectivity. The medians were calculated across subjects for each considered parcellation for the phase oscillator (red) and the neural mass model (blue) as indicated in the legend together with the fraction of the explained variance. The symbols stand for the individual parcellations from Table 1. (**D**) Cumulative amount of explained variance in the group-averaged graph-theoretical measures as a function of the number of included PCs. (**E**) Fraction of the interparcellation variance of the goodness-of-fit being explained by the (multivariate) linear regression model as a function of the number of PCs included in the model. Other abbreviation: a.u. = arbitrary unit, cumul. = cumulative, expl. = explained, var. = variance.

To understand the effects observed at the group level, the patterns of the extracted graph-theoretical statistics across parcellations (Figure 2, Figure 3, median values) were compared with one another and with those obtained for the goodness-of-fit of both models (Figure 6A, median values). Significant correlations were observed for some of the tested combinations, which are shown in Figure 7A. This in particular concerned the correlations of the inverted number of parcels with the subject medians of the modularities and clustering coefficients of both the empirical SC and FC, the scale parameters of the degree distributions of the empirical FC, and the correlations between empirical SC and FC (Figure 7A, top row/first column). In such a way the dependencies of these measures on granularity were demonstrated, which were already observed in Figure 4. Furthermore, the scale parameters of the degree distributions of the empirical SC and the structure-function relationship between the empirical SC and FC exhibited significant correlations with the fitting results for both models. Interestingly, the modularity of the empirical FC significantly anticorrelated with fitting results for the neural mass model (i.e., smaller modularity implies better fitting), but not for the phase model (Figure 7A).

We thus found that the network properties of the empirical connectomes (Figure 2, Figure 3) and the quality of the model validation as given by the goodness-of-fit of the simulated FC to the empirical FC (Figure 6) in some cases demonstrated a pronounced and significant correlation with one another across parcellations (Figure 7A). To quantify this relationship further, we combined principal component analysis with ordinary least squares linear regression to take into account the contributions from all graph-theoretical statistics; see Materials and Methods (*Analysis*) for details of this analysis. The first principal component (PC1) extracted from the group-averaged graph-theoretical statistics was found to explain 48% of the variance in the data variables across parcellations (Figure 7D), and the signs of its relative loadings (Figure 7B) were in accordance with previous results (see e.g., Figure 7A). Subsequently, we regressed the PC1 scores with the medians of the goodness-of-fit calculated across subjects for every brain atlas. We found that this PC explained about 19% and 49% of the interparcellation variance in the goodness-of-fit for the phase oscillators and the neural mass models, respectively (Figure 7C). We again observed stronger contribution of empirical data to the fitting results of the neuronal mass model; see also Figure 4O, P.

The second principal component (PC2) explained an additional 35% of the variance in the data variables (Figure 7D). We included this component in the linear regression model, which made it multivariate. This improved the association between the data variables and the goodness-of-fit to 77% and 81% of explained variance for the phase oscillator and neural mass model, respectively (Figure 7E). Including more principal components in the linear regression model led to an even better explanation of the goodness-of-fit variance by the empirical data (Figure 7D, E). Note, however, that using too many PCs in the regression may lead to an overfitting for the considered 19 parcellations. Finally, we investigated the effect of the granularity on these results by regressing this effect out of all the quantities used in this investigation while following the same procedure as described above. The results of this inquiry are shown in Figure S7 in the Supplementary Results, and they demonstrate that after the removal of the granularity effect already the first principal component sufficed to get approximately the same associations between the data variables and the goodness-of-fit as observed in Figure 7 for two PCs. Also the difference between models was inverted and reduced.

With these results, we demonstrated that most of the interparcellation variation observed in the modeling results at the group level (Figure 6A) could be explained by the network properties of and the relationship between empirical SC and FC used to inform and validate the models. Furthermore, we showed which metrics derived from the empirical connectomes contributed positively and negatively to the goodness-of-fit of the simulated FC produced by the model to the empirical FC (Figure 7B). Lastly, our results confirm that the parcellation exerts an influence on the graph-theoretical measures and the goodness-of-fits that can only be partially explained by the granularity. This especially becomes evident when considering the high PC1 loading of the inverse of the number of parcels in relation to the relatively low association of this PC with the modeling results (Figure 7B, D); see also Figure S7 in the Supplementary Results, where the granularity was regressed out.

### Interindividual Differences of Empirical Connectomes and Modeling Results

As shown above, the group averages of the graph-theoretical statistics and the modeling results obtained using different brain atlases are tightly related to one another (Figure 7). Nevertheless, as dynamical whole-brain models seem to be a promising model-based approach for studying interindividual differences (Ritter et al., 2013; Sanz-Leon et al., 2015; Zimmermann et al., 2018), we investigated whether the within-parcellation, between-subject variances observed in our modeling results could also be attributed to variations of the data variables extracted from the empirical SC and FC. To do so, we adopted the approach from the previous section, where, for each individual parcellation, we built a separate dataset containing the corresponding graph-theoretical network properties; see Materials and Methods (*Analysis*) for details. Using this dataset, we initially checked how individual empirical graph-theoretical statistics correlated with the interindividual variability of the goodness-of-fit, and found no clear correspondences except for the structure-function relationship *ρ*_*SC*,*FC*_ (Figure S8). It is interesting to observe here that *ρ*_*SC*,*FC*_ correlated negatively with the goodness-of-fit of the models to the empirical data for most of the considered parcellations. Given that this bivariate approach did not yield positive results in the form of clear (anti)correlations for the investigated network metrics, we resorted to multivariate analyses.

As before, we calculated the PC1 of the consequent dataset of z-scored individual data variables (network properties) and subsequently regressed the PC1 scores with the corresponding goodness-of-fits of the model across individual subject-session pairs for every one of the considered brain atlases. The obtained results showed that the amount of variance in the modeling results across subjects explained by PC1 was low (<3%; see Figure S9A in the Supplementary Results), even though the data variables extracted using different parcellations exhibited similar covariations as reflected by the PC1 loadings and corresponding correlations, which exhibited some form of clustering (Figure S9B–C). Because of the weak explanatory power observed at this approach (Figure S9A), the used methodology based on the principal component analysis of network properties of empirical connectomes might be inappropriate to assess interindividual differences in the model validation.

**Figure 8.**
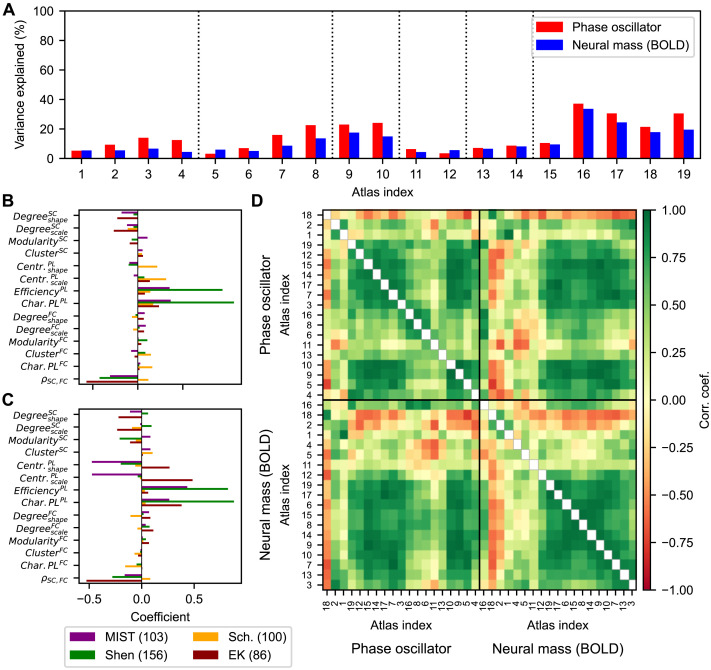
Relationship between the interindividual variations of the empirical graph-theoretical metrics and modeling results for different parcellations. (**A**) Amounts of within-parcellation, between-subject variance in the modeling results (goodness-of-fit to empirical data) being explained via multivariate ordinary least squares linear regression utilizing the z-scored graph-theoretical statistics of the empirical connectomes per parcellation. Modeling results were sampled by using the systems of coupled phase oscillators (red) and neural mass models (blue). (**B**–**C**) Regression coefficients corresponding to the data variables (network properties) depicted in Figure 2 and Figure 3 for four selected brain parcellations as indicated in the legend and for the phase oscillators (B) and the neural mass models (C) leading to the regression results in panel A. The abbreviations MIST (103), Shen (156), Sch. (100), and EK (86) correspond to the parcellations in Table 1 and in panel A with indices 3, 10, 11, and 16, respectively. (**D**) Pearson correlation coefficients across the regression coefficients per pair of brain parcellation and model type. Table 1 contains the parcellation information corresponding to the atlas indices. Abbreviations: coef. = coefficient, corr. = correlation.

We therefore employed a different approach, where the z-scored data variables representing the network properties of empirical SC and FC were directly regressed with the z-scored goodness-of-fits of the models across individual subjects via multivariate ordinary least squares regression. The regression results obtained for individual parcellations indicated a variable amount of explained between-subject variance in the goodness-of-fit for different parcellations (Figure 8A). The strongest influences of the empirical connectomes on the interindividual variations in the goodness-of-fit were observed for the von Economo-Koskinas, AAL, and Brainnetome atlases (indices 16, 17, and 19 in Figure 8A, respectively), which however still did not exceed 40% of explained variance. For other parcellations based on, for example, the Schaefer or Harvard-Oxford atlases (indices 11–14 in Figure 8A), the results of the model fitting for an individual subject practically did not depend on the network properties of the used empirical connectomes. Interestingly, in most cases the phase oscillators exhibited a somewhat stronger dependence on the considered data variables (Figure 8A, red bars), which contrasts with the interparcellation variation of the medians (Figure 7E). We observed low consistency between parcellations and between models regarding the regression coefficients assigned to the corresponding data variable by this multivariate regression analysis (Figure 8B–C). This is reflected by the Pearson correlations across the coefficients per model and parcellation pair illustrated in Figure 8D, which shows a clustering that is inconsistent across models.

Taken together, these results demonstrated that the contributions of the graph-theoretical statistics derived from the empirical connectomes to the interindividual differences in the modeling results were limited.

### Network Properties of Simulated Functional Connectomes

We established that between-parcellation variances in the model fitting results could largely be explained by the variation of the network properties taken from the empirical SC and FC (Figure 7). However, we also found that such a relationship was hardly applicable to the explanation of the intraparcellation, between-subject variations. In this case, for any parcellation, the interindividual differences in the goodness-of-fit only weakly to moderately correlated with the graph-theoretical properties of empirical networks for individual subjects (Figure 8).

**Figure 9.**
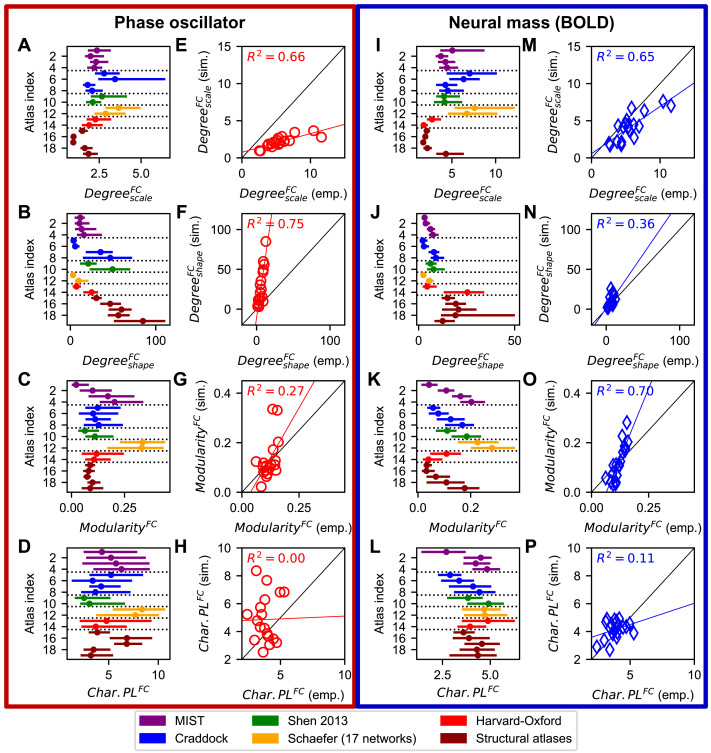
Relationship between the graph-theoretical statistics of empirical and simulated FC matrices at the group level. Network properties of the simulated FCs providing the best fits to the empirical FC and the scatterplots of the corresponding median values calculated across subjects are illustrated for phase oscillator model (A–F) and neural mass model (G–L). The shape (A, E, I, M) and scale (B, F, J, N) parameters of the degree distributions, the modularities (C, G, K, O) and the characteristic path lengths (D, H, L, P) are depicted for the parcellations in Table 1, where dots and lines in panels A–D and I–L depict the medians and interquartile ranges across subjects, respectively. Symbols, colored lines, and black lines in the scatterplots (E–H) and (M–P) of the simulated network metrics versus empirical ones stand for individual parcellations, regression lines, and the diagonal *x* = *y*, respectively.

Here we evaluate how similar the empirical FC matrices were in terms of the graph-theoretical statistics to the simulated ones that provided the best fits based on Pearson’s correlation. To do so, the simulated FC matrices were subjected to the same graph-theoretical analyses as the empirical FCs; see Figure 9A–D, I–L for results. The medians of the network properties calculated across subjects for the empirical and simulated FCs were correlated with each other over all considered parcellations. The results showed that relationships between the network properties of the empirical and simulated FCs existed, which indicated that the models on average preserved most of the considered network properties of the empirical functional connectome; only the characteristic path length exhibited low coefficients of determinations for both models (Figure 9E–H, M–P). The results for the clustering coefficient have not been shown in Figure 9 as they resembled those of the modularity. We also found that the empirical and simulated functional networks agreed with each other to very different extents for the two considered models except for the shape parameter of the degree distribution (Figure 9E, M). More variance in the scale parameters of the degree distributions of the simulated FC across parcellations could be explained by that of the empirical FC when the phase oscillators rather than the neural mass models were used for the generation of the former (Figure 9F, N). The opposite is true for the modularity and characteristic path length; here, the neural mass model led to more explained variance (Figure 9G–H, O–P). From these results, we can conclude that the accuracy of the transformation of the empirical SC to simulated FC by the considered dynamical whole-brain models can depend on the model used for the simulation of the local mean activity of the brain regions. These findings furthermore indicated that, even though different models may lead to comparable goodness-of-fits (Figure 6C), the correspondence of the network structures of the simulated FCs to those of the empirical ones may vary considerably across models.

**Figure 10.**
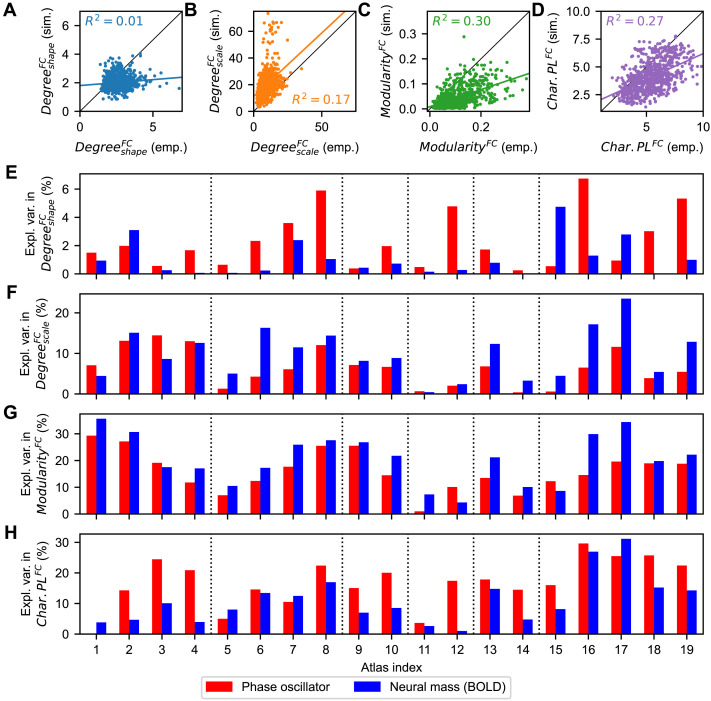
Relationship between the graph-theoretical statistics of empirical and simulated FC matrices at the subject level. (**A**–**D**) Scatterplots of the shape (A) and scale (B) parameters of the degree distributions, the modularities (C), and the characteristic path length (D) of the empirical and simulated (by neural mass model) FCs within a single parcellation as given by the von Economo-Koskinas atlas (index 16 in Table 1). Every dot represents a subject-session pair, the colored lines depict the ordinary least squares linear regression solution, and the black lines correspond to *x* = *y*. (**E**–**H**) Proportion of intersubject variance of the network properties of the best-fit simulated FCs generated by the phase oscillators (red) and the neural mass model (blue) that is explained by the network properties of the empirical FCs for a given parcellation indicated on the horizontal axes. Table 1 contains the parcellation information corresponding to the atlas indices used in the plots. Other abbreviations: expl. = explained, var. = variance.

Finally, we investigated how the latter analysis performed at the level of individual subjects and individual parcellations. Hence, we correlated the network properties derived from the empirical and simulated FCs across subjects for each individual parcellation. The obtained results, illustrated in Figure 10A–D, revealed that the highest correspondences between the network properties of the empirical and simulated FC could be found for the modularity and characteristic path length (Figure 10G–H). No general patterns could be found as to which model led to higher explained variances between empirical and simulated FC (Figure 10E–H). Still, we observed relatively large deviations of the explained variance between the two considered models for the individual parcellations, where the largest differences between the models could reach around 20% of explained variance (Figure 10E–H, differences between red and blue bars).

These results show that network properties of the empirical and simulated FCs could be a good measure of the model validation, and allow us to distinguish different models at the level of individual subjects (Figure 10) as well as at the group level (Figure 9). This seemed not to be the case for the correlative model fitting, where the models were practically indistinguishable at the group level and could be differentiated only at the subject levels. The latter claim can be seen in the amount of variance in the goodness-of-fit that is explained by the network properties derived from the empirical data when comparing between- and within-parcellation variations (see Figure 7, Figure 8).

## DISCUSSION

In this study, we used a selection of 19 parcellations constructed through 10 different approaches. They were selected with an attempt to balance between parcellations derived from functional data, comprising the atlases described by Craddock et al. (2012), Shen et al. (2013), Schaefer et al. (2018), and Urchs et al. (2019), and structural information, constituting the other parcellations included in Table 1. Furthermore, the investigated parcellations were compiled using distinct methodologies such as boundary detection algorithms, histological stainings, and diverse clustering approaches (see the Supplementary Method for details). While more brain parcellations are available in the literature and were used for data-driven analyses (Dadi et al., 2020; Messé, 2020; Schaefer et al., 2018), the tested parcellations and the variation regarding the number of parcels in them are representative for the state-of-the-art brain parcellations, and can support the derived conclusions concerning the reported relationship between the model simulation results and the empirical data.

### Influence of Parcellation on Graph-Theoretical Statistics and Goodness-of-Fit

Significant (anti)correlations were found across parcellations when comparing the parcellation granularity with individual graph-theoretical statistics and the goodness-of-fit of the whole-brain models (Figure 7). This clearly evidenced the granularity substantially affecting the network properties of the empirical FC and SC and the model fitting results regardless of the method used to construct the parcellation. Still, as the parcellation varied, graph-theoretical statistics as well as the goodness-of-fit exhibited pronounced variations (Figure 2, Figure 3, Figure 4, Figure 6) that persisted after we corrected for the effect of the granularity (Figure S3, Figure S4, Figure S6, Figure S5). We were not able to reliably distinguish between results derived from e.g. functionally and structurally derived parcellations, even after the granularity correction was performed. Hence, as parcellation-induced variances in the goodness-of-fit were shown to be related to the variations in the empirical SC and FC, the question still remains how the parcellations induce the pronounced differences in the graph-theoretical statistics.

The reported parcellation-induced variances emphasize the importance of a well-advised selection of the parcellations in region-based neuroimaging studies using graph theory or whole-brain models to analyse the data. A recent study by Messé (2020) already showed this to be true when examining the structure-function relationship of the brain from a statistical perspective. Also studies by Wang et al. (2009) and Zalesky et al. (2010) demonstrated the prominent influence the brain parcellation may have on the network properties of the empirical FC and SC, respectively. Our study added further modern graph-theoretical measures to the analysis for both empirical SC and FC as well as simulated FC. In sum, these findings can complement other considerations (e.g., the biological interpretation of the atlas) in the selection of the proper parcellation for the study at hand. After all, the question concerning an optimal parcellation is a difficult problem given many possible parcellation techniques and optimization criteria.

### Important Factors With Respect to Model Fitting

We found that most of the interparcellation variance in the goodness-of-fit at the group level could be explained by the graph-theoretical statistics derived from the empirical SC and FC (Figure 7). By examining the PC1 and PC2 loadings in Figure 7B, the graph-theoretical measures associated with a high goodness-of-fit can be identified. Here, the PC1 loadings clearly reflected the effect of granularity in the graph-theoretical statistics and demonstrated that a finer granularity leads to a lower goodness-of-fit. The loadings of PC2, which explained a large amount of variance in the modeling results for both models, did not exhibit such a general relation. The parameters of the degree and closeness centrality distributions as well as the global efficiency are heavily loaded onto this PC. Here, the shape parameters of all the fitted gamma distributions exhibited negative loadings, implying that a small shape parameter leads to a high goodness-of-fit (see also Figure S7). Given Equation 1 and Figure S1, this implies that the modeling workflow prefers that most nodes have a low centrality and a select few nodes have a high centrality for the empirical SC as well as FC, because then the density is high close to 0 and decreases with incrementing degree. The positive PC2 loading of the global efficiency, furthermore, implies that the whole-brain models can replicate the functional networks better if the structural networks facilitate the integration of signals.

The network architecture of the brain itself is believed to comprise a multilevel modular structure and a heterogeneity with respect to the degree of individual nodes (Avena-Koenigsberger, Misic, & Sporns, 2018; van den Heuvel & Sporns, 2019). Although the modularity did not exhibit a strong relationship with the goodness-of-fit other than their shared dependence on the granularity (Figure 7, Figure S7), our results show that dynamical whole-brain models indeed favor such a heterogeneity in the degree distribution. After all, the goodness-of-fit was ameliorated by a higher diversity with regard to the degree distribution in the SC and FC (as illustrated in Figure S10A–E).

### Within-Parcellation, Between-Subject Variances, and the Personalization of Whole-Brain Models

Previous studies suggested that dynamical whole-brain models are able to simulate the resting-state brain activity on a personalized level (Bansal et al., 2018; Deco et al., 2017; Ritter et al., 2013; Sanz-Leon et al., 2015; Zimmermann et al., 2018). How this personalization is achieved is not known. In this study, we have provided evidence that interindividual differences in the goodness-of-fit do not reliably relate to the subject-specific deviations in the graph-theoretical measures (Figure 8, Figure S9). In addition, we have shown that the network structures of the simulated FC map onto those of the empirical FC when considering group averages, but not within-parcellation, interindividual variances (Figure 9, Figure 10). Taken together, the personalization of whole-brain models does not seem to use subject-specific deviations in the network properties. How personalization of whole-brain models then actually is achieved requires further investigation.

To account for the interindividual variations of the modeling results, other data variables may for example be considered out of the class of the considered network properties. In such investigations, special attention must be paid to the limitations in the reconstruction of the structural connectome. Studies namely have shown substantial amounts of inaccuracies (e.g., false positives or negatives) infecting the empirical SC when it is extracted from dwMRI data (Bassett, Brown, Deshpande, Carlson, & Grafton, 2011; Lindquist, 2020; Maier-Hein et al., 2017; Schilling et al., 2019; Sotiropoulos & Zalesky, 2019). These inaccuracies can have a systematic effect on the network properties of the empirical SC (Zalesky et al., 2016). In order to reduce these inaccuracies, the whole-brain tractography should be calculated with high density by state-of-the-art techniques, as we did in this study, which can enhance its reproducibility (Roine et al., 2019).

The patterns of the intersubject differences in the graph-theoretical statistics and the modelling results may vary across parcellations (Figure 5, Figure 6B), which implies that the network structures of the empirical connectomes and the modeling results depend on the used parcellation also at the level of individual subjects. This is a relevant implication as it may have consequences for computational modeling studies investigating clinical traits (Cabral, Hugues, Kringelbach, & Deco, 2012; Saenger et al., 2017). Observed differences between groups and individual subjects may deviate when another parcellation is used and may therefore reflect artefacts induced through the use of a particular parcellation rather than actual deviations in the structure-function relationship of distinct cohorts, as also discussed by Betzel and Bassett (2017).

### Perspectives and Outlook

Further brain parcellations, datasets, models, and (graph-theoretical) analyses variations might be considered to verify and confirm the obtained results, especially when more computationally powerful resources become available. In the end, the simulations and optimizations of dynamical whole-brain models are computationally costly. The computational costs also inhibit the estimation of biases in the model simulation results via, for example, null models. Future studies should be devoted to devising strategies that could estimate these biases without a full evaluation of the model dynamics through simulations.

Related to these computational costs is the notion that our results can contribute to the development of informed expectations concerning the quality of the model validation for a given brain parcellation. For this, a few network properties of the empirical connectomes calculated for this parcellation can be examined before running time- and resource-consuming model simulations. Additionally, this concept may be exploited to distinguish between data-induced and model-induced deviations in the modeling results. Such an investigation may estimate to what extent the empirical data already predicted the differences in modeling results between, for example, healthy and clinical cohorts; the contribution of the model is consequently represented by the remaining between-group variance.

Finally, the inaccurate mappings of empirical SC to simulated FC by both tested models for local, mean-field activity highlight their current limitations with respect to the replication of empirical resting-state brain dynamics. How well other types of models can replicate the empirical FC on the basis of the empirical SC remains to be seen and should be investigated further. Such an investigation would typically comprise the application of the framework of this study to other model types such as the Jansen-Rit model (Jansen & Rit, 1995; Jansen, Zouridakis, & Brandt, 1993), the (reduced) Wong-Wang model (Deco & Kringelbach, 2014; Hansen, Battaglia, Spiegler, Deco, & Jirsa, 2015; Wong & Wang, 2006), different types of limit-cycle oscillators (Deco et al., 2018; Deco et al., 2017; Ghosh, Rho, McIntosh, Kötter, & Jirsa, 2008), and a more recently developed neural mass model that incorporates plasticity dynamics (Abeysuriya et al., 2018). Taken together, this implies that, even though the tested models yield results that are related to the empirical data in terms of more than one statistic, they are far from perfect and hence there is room for improvement.

## ACKNOWLEDGMENTS

We thank M. Kollmann for his consultation on the manuscript and S. Zhang for his consultation on the parcellation image processing. This study was made possible through the Portfolio Theme Supercomputing and Modeling for the Human Brain of the Helmholtz Association (https://www.helmholtz.de/en), and through the European Union’s Horizon 2020 Research and Innovation Program. The funders had no role in study design, data collection and analysis, decision to publish, or preparation of the manuscript. Also, the authors gratefully acknowledge the computing time granted through JARA on the supercomputer JURECA at Forschungszentrum Jülich.

## SUPPORTING INFORMATION

Supporting information for this article is available at https://doi.org/10.1162/netn_a_00202. We provide three types of supporting information. The **Supplementary Method** contains a summary on the construction of the parcellation schemes that we used in our study, i.e. what methodology was applied to what type of data to construct them. In addition, it discloses how each brain parcellation image was modified in order to enhance the comparability between parcellations. The **Supplementary Data Sheet** includes depictions of the empirical SC, PL and FC matrices acquired through the use of the same brain parcellations. In addition, the data sheet displays statistics derived from the empirical connectomes. Finally, the **Supplementary Results** contains figures that were used to support the main results.

## AUTHOR CONTRIBUTIONS

Justin Domhof: Conceptualization; Data curation; Formal analysis; Investigation; Methodology; Software; Validation; Visualization; Writing – original draft; Writing – review & editing. Kyesam Jung: Data curation; Methodology; Software; Writing – review & editing. Simon Eickhoff: Conceptualization; Funding acquisition; Project administration; Supervision; Validation; Writing – review & editing. Oleksandr Popovych: Conceptualization; Funding acquisition; Methodology; Project administration; Resources; Supervision; Validation; Writing – original draft; Writing – review & editing.

## FUNDING INFORMATION

Simon Eickhoff, Horizon 2020 (https://dx.doi.org/10.13039/501100007601), Award ID: 785907. Simon Eickhoff, Horizon 2020 (https://dx.doi.org/10.13039/501100007601), Award ID: 945539. Simon Eickhoff, Horizon 2020 (https://dx.doi.org/10.13039/501100007601), Award ID: 826421.

## TECHNICAL TERMS

Structure-function relationship: The correspondence between structural and functional connectivity.
Structural connectivity: Reflection of the physical, anatomical connections throughout the brain.
Functional connectivity: Reflection of synchronized coactivations throughout the brain.
Dynamical whole-brain model: A model that simulates brain activity on the basis of the structural connectivity in order to replicate the functional connectivity.
Brain atlas or parcellation: A description of the delineation of the brain into distinct regions.
Granularity: The number of brain regions in a parcellation.
Empirical structural connectivity: Structural connectivity derived from (dwMRI) data.
Empirical functional connectivity: Functional connectivity derived from (fMRI) data.
Simulated functional connectivity: Functional connectivity simulated by a dynamical whole-brain model.
Goodness-of-fit: The maximized fit between the empirical functional connectivity and the functional connectivity simulated by a dynamical whole-brain model.
