# Supplementary material for: Parcellation-induced variation of empirical and simulated brain connectomes at group and subject levels
Source: Netw Neurosci. 2021 Aug 30;5(3):798–830. doi: 10.1162/netn_a_00202 (PMC8567834; doi:10.1162/netn_a_00202)
Supplement: Supplementary file 1 [file netn-05-798-s001.pdf]

Domhof, J. W. M., Jung, K., Eickhoff, S. B. & Popovych, O. V. (2021). Supporting information for "Parcellation-induced variation of empirical and simulated brain connectomes at group and subject levels." *Network Neuroscience.* Advance publication. https://doi.org/10.1162/netn_a_00202

Supplementary Data Sheet

Every datasheet contains nine plots. The top row contains examples of the structural connectivity, path length and functional connectivity matrices (from left to right). These examples were constructed using the same subject of the HCP dataset. Modularity and modular structure were calculated from the structural and functional connectivity matrices and subsequently matrix rows and columns were sorted on the basis of these results. The modularity index and the number of modules are also displayed within the structural and functional connectivity matrices and the global efficiency calculated from the PL matrix is displayed in that matrix. The middle row contains the densities of the individual elements of the corresponding matrices calculated across the 200 subjects from the HCP dataset used in this study. The bottom row shows (from left to right) the degree distribution of the empirical SC matrix, the closeness centrality of the empirical PL matrix and the degree distribution of the empirical FC matrix. These distributions were calculated across the 200 subjects from the HCP dataset used in this study. In addition, the gamma and normal distributions that best fitted the empirical distribution at hand are drawn into the plots. The legends explain which line corresponds to which distribution. Additionally, they also display the Kolmogorov-Smirnov statistic (in parentheses), which characterises the quality of the fit of the parametric distribution to the empirical data as reflected by the distance between the cumulative distribution functions. A lower value of this statistic implies a better fit.

MIST

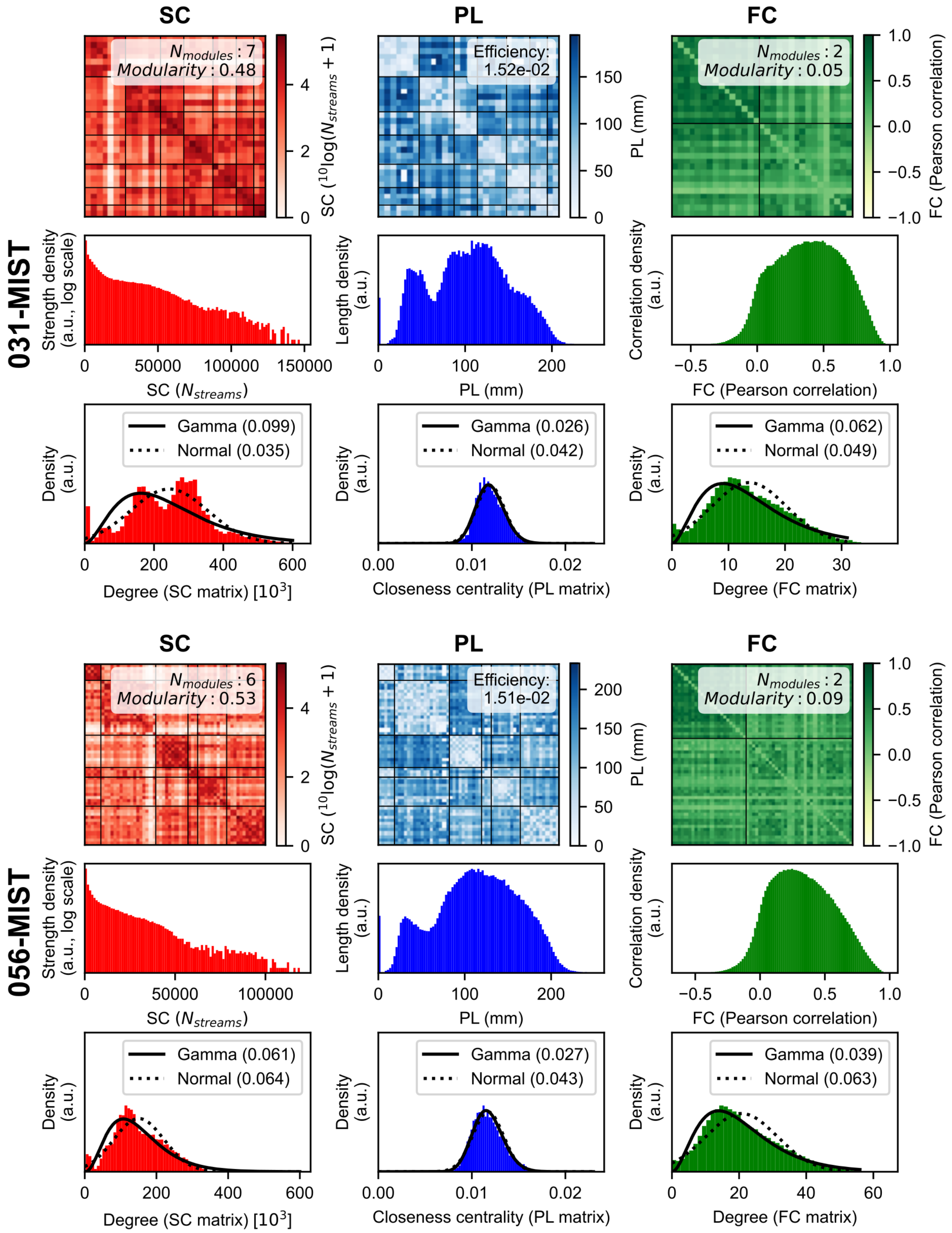
SC
PL
FC
$N_{modules}: 7$
$Modularity: 0.48$
Efficiency:
1.52e-02
$N_{modules}: 2$
$Modularity: 0.05$
SC ($^{10}\log(N_{streams} + 1)$)
PL (mm)
FC (Pearson correlation)
031-MIST
Strength density (a.u., log scale)
SC ($N_{streams}$)
Length density (a.u.)
PL (mm)
Correlation density (a.u.)
FC (Pearson correlation)
Gamma (0.099)
Normal (0.035)
Degree (SC matrix) [10^3]
Gamma (0.026)
Normal (0.042)
Closeness centrality (PL matrix)
Gamma (0.062)
Normal (0.049)
Degree (FC matrix)
Density (a.u.)
SC
PL
FC
$N_{modules}: 6$
$Modularity: 0.53$
Efficiency:
1.51e-02
$N_{modules}: 2$
$Modularity: 0.09$
SC ($^{10}\log(N_{streams} + 1)$)
PL (mm)
FC (Pearson correlation)
056-MIST
Strength density (a.u., log scale)
SC ($N_{streams}$)
Length density (a.u.)
PL (mm)
Correlation density (a.u.)
FC (Pearson correlation)
Gamma (0.061)
Normal (0.064)
Degree (SC matrix) [10^3]
Gamma (0.027)
Normal (0.043)
Closeness centrality (PL matrix)
Gamma (0.039)
Normal (0.063)
Degree (FC matrix)
Density (a.u.)

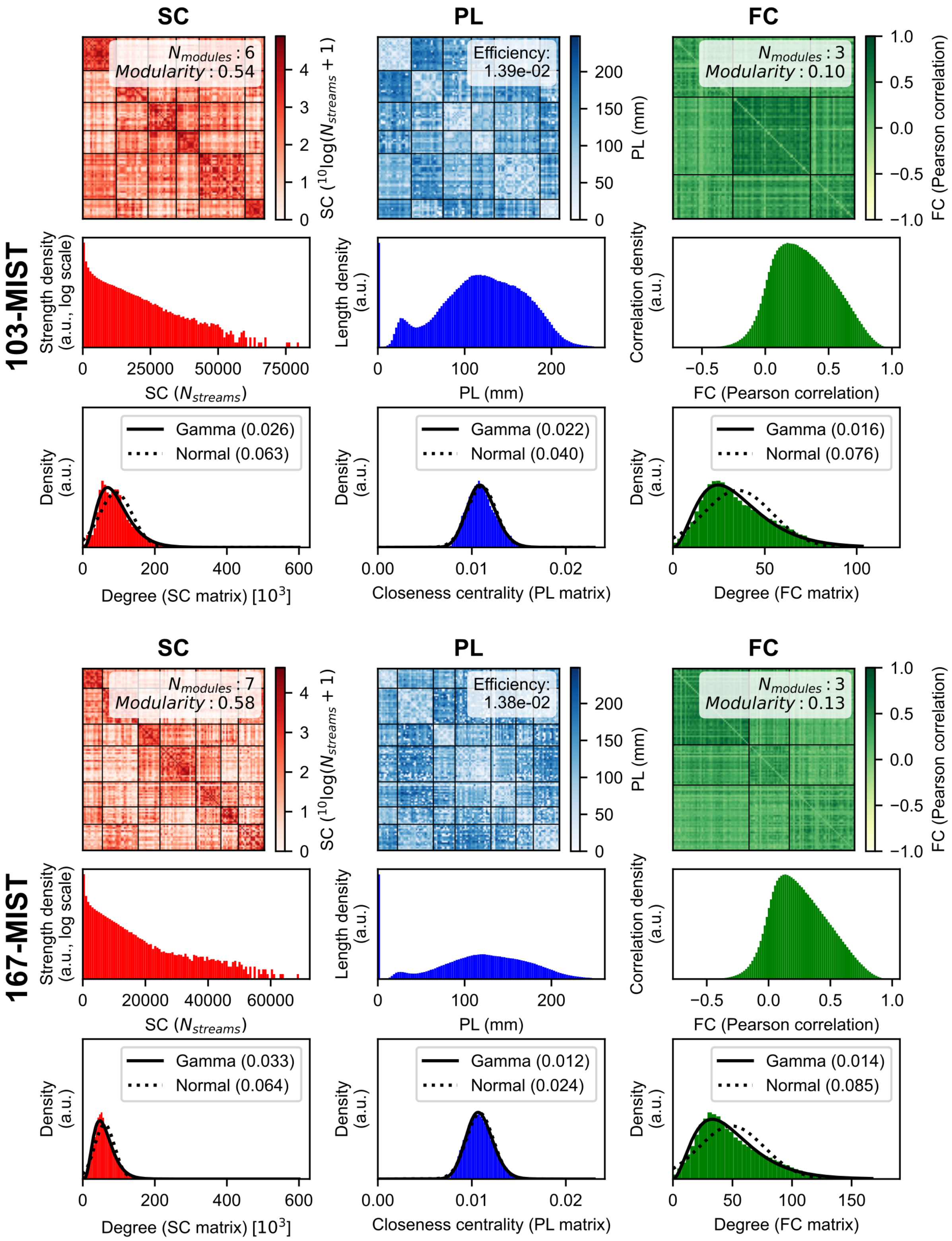
SC
PL
FC
$N_{modules}$: 6
$Modularity$: 0.54
Efficiency:
1.39e-02
$N_{modules}$: 3
$Modularity$: 0.10
SC (^{10}log($N_{streams}$ + 1)
PL (mm)
FC (Pearson correlation)
103-MIST
Strength density
(a.u., log scale)
SC ($N_{streams}$)
Length density
(a.u.)
PL (mm)
Correlation density
(a.u.)
FC (Pearson correlation)
Density
(a.u.)
Gamma (0.026)
Normal (0.063)
Degree (SC matrix) [10^3]
Gamma (0.022)
Normal (0.040)
Closeness centrality (PL matrix)
Gamma (0.016)
Normal (0.076)
Degree (FC matrix)
SC
PL
FC
$N_{modules}$: 7
$Modularity$: 0.58
Efficiency:
1.38e-02
$N_{modules}$: 3
$Modularity$: 0.13
167-MIST
Gamma (0.033)
Normal (0.064)
Gamma (0.012)
Normal (0.024)
Gamma (0.014)
Normal (0.085)

CraddockSCorr2Level

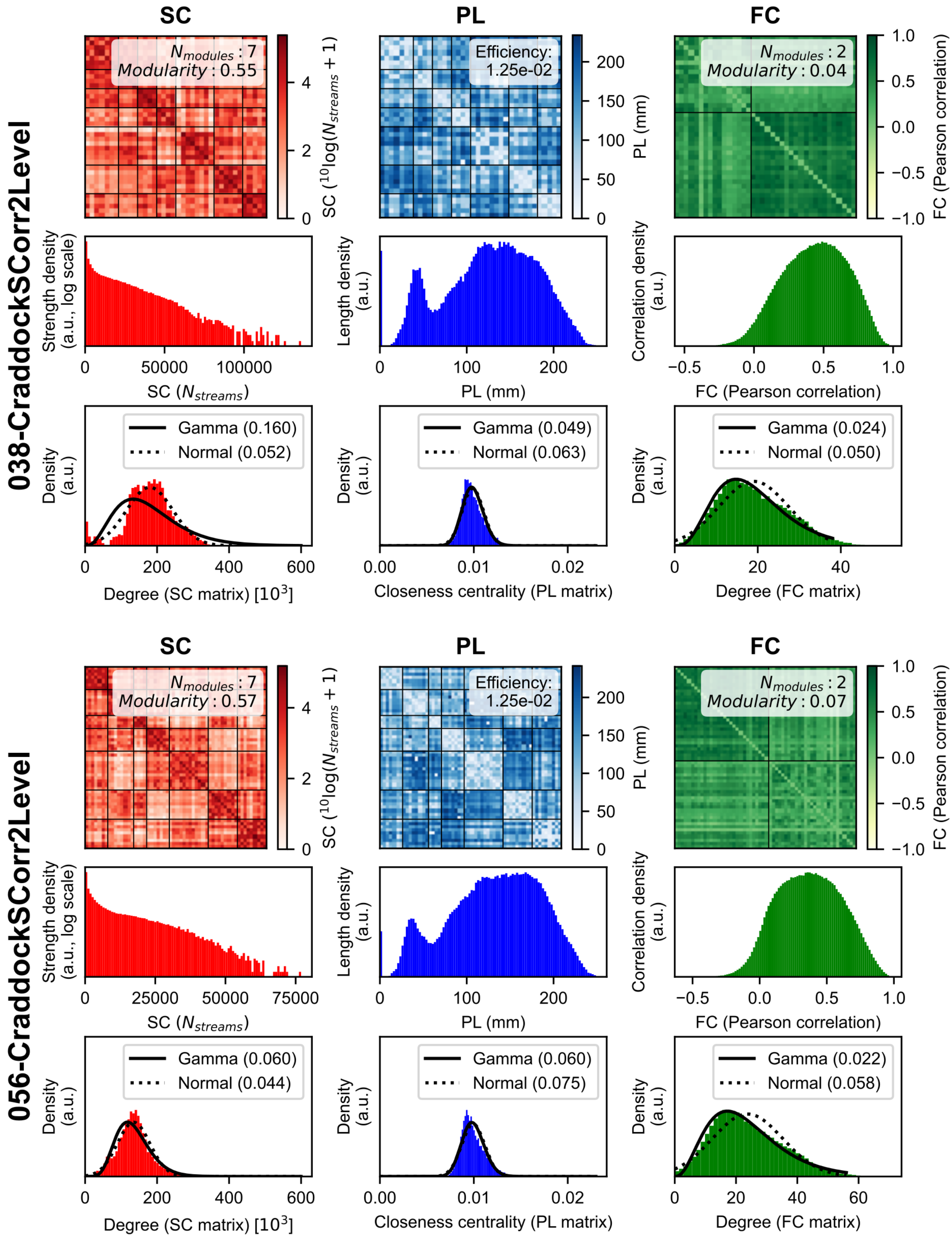

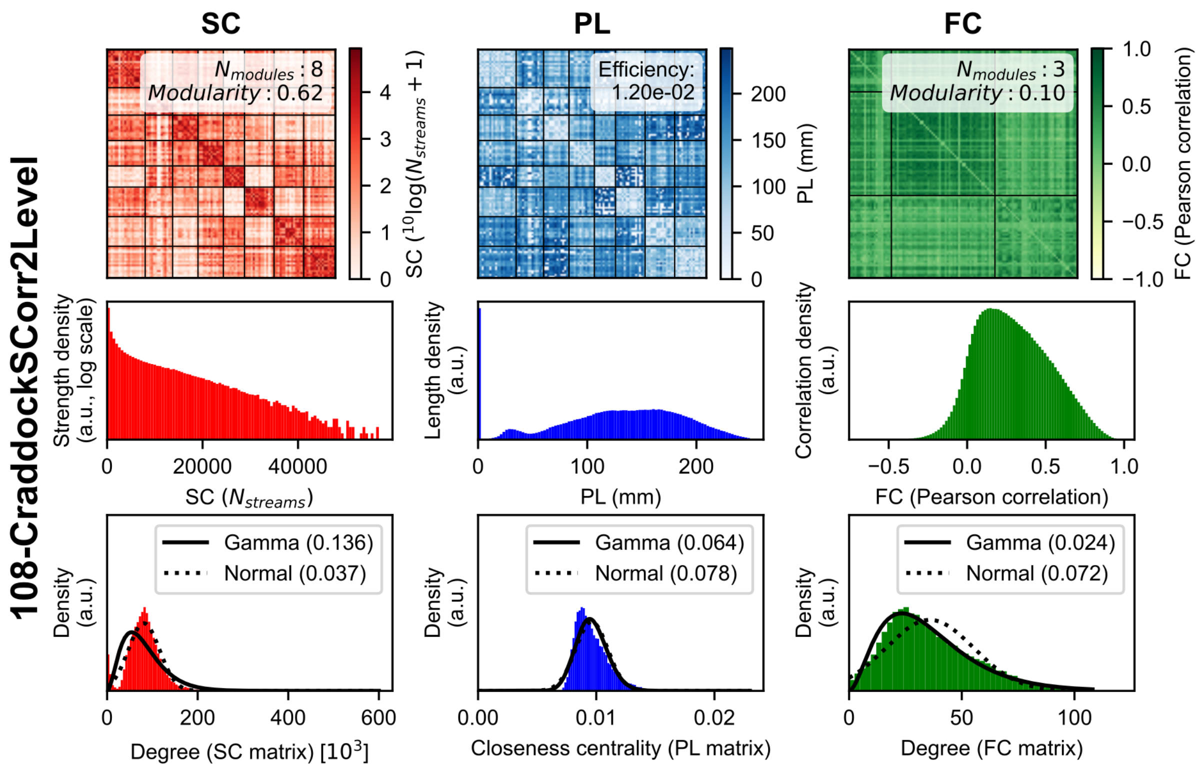
108-CraddockSCorr2Level
SC
PL
FC
$N_{modules}: 8$
$Modularity: 0.62$
Efficiency:
1.20e-02
$N_{modules}: 3$
$Modularity: 0.10$
SC ($^{10}\log(N_{streams} + 1)$)
PL (mm)
FC (Pearson correlation)
Strength density
(a.u., log scale)
SC ($N_{streams}$)
Length density
(a.u.)
PL (mm)
Correlation density
(a.u.)
FC (Pearson correlation)
Gamma (0.136)
Normal (0.037)
Density
(a.u.)
Degree (SC matrix) [10^3]
Gamma (0.064)
Normal (0.078)
Closeness centrality (PL matrix)
Gamma (0.024)
Normal (0.072)
Degree (FC matrix)

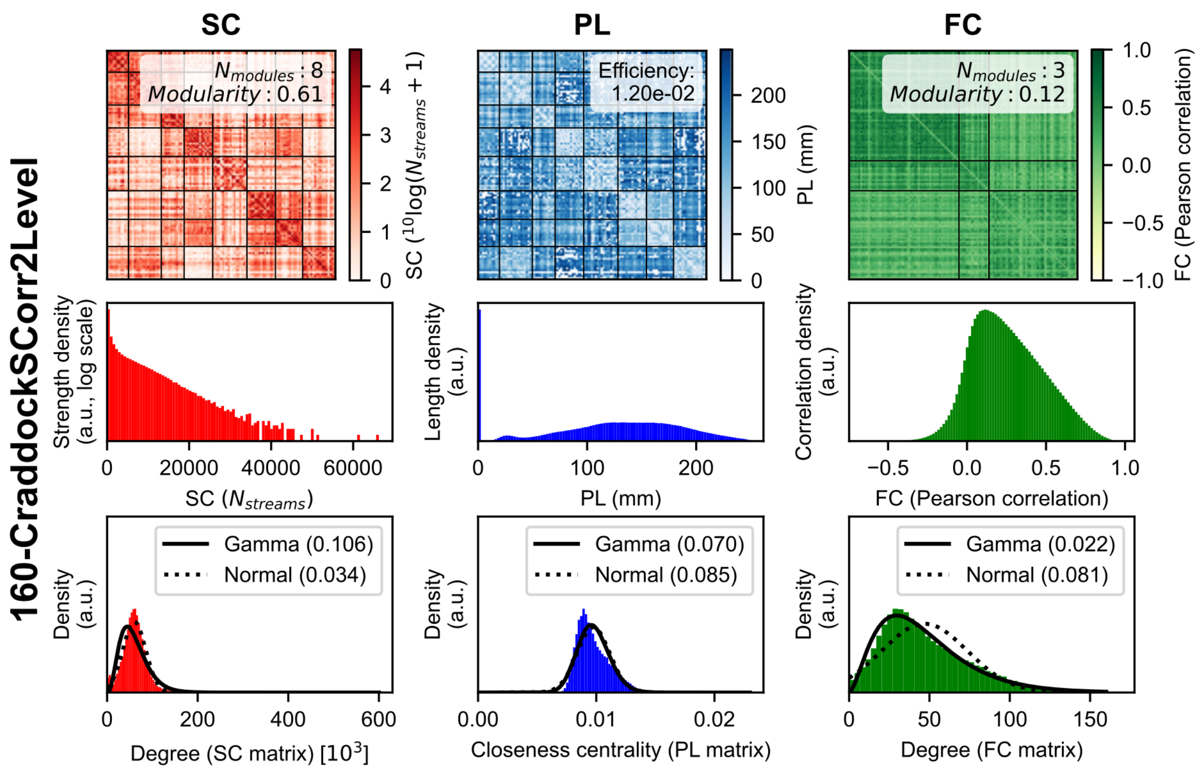
160-CraddockSCorr2Level
SC
PL
FC
$N_{modules}: 8$
$Modularity: 0.61$
Efficiency:
1.20e-02
$N_{modules}: 3$
$Modularity: 0.12$
SC ($^{10}\log(N_{streams} + 1)$)
PL (mm)
FC (Pearson correlation)
Strength density
(a.u., log scale)
SC ($N_{streams}$)
Length density
(a.u.)
PL (mm)
Correlation density
(a.u.)
FC (Pearson correlation)
Gamma (0.106)
Normal (0.034)
Density
(a.u.)
Degree (SC matrix) [10^3]
Gamma (0.070)
Normal (0.085)
Closeness centrality (PL matrix)
Gamma (0.022)
Normal (0.081)
Degree (FC matrix)

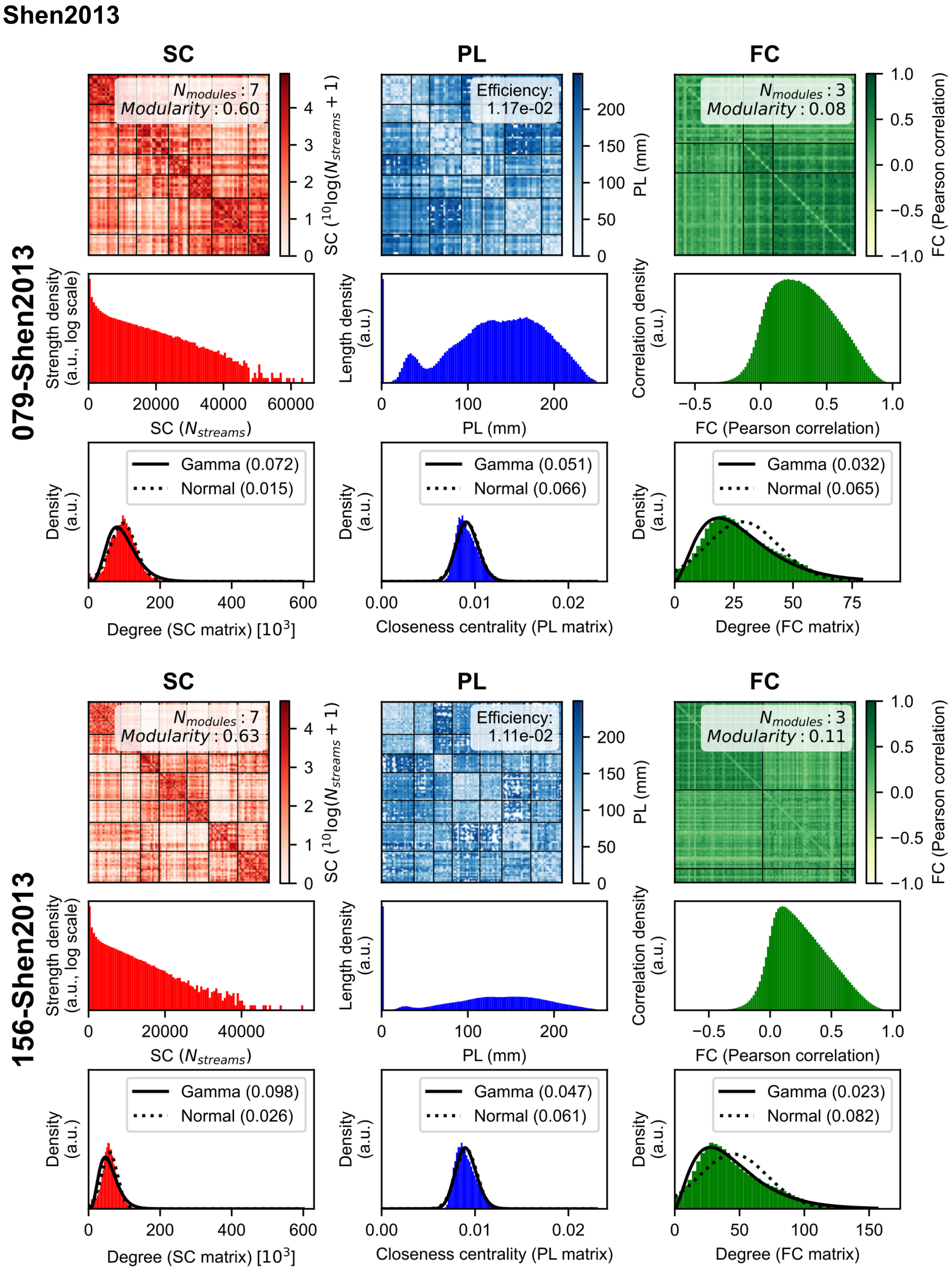
Shen2013
079-Shen2013
SC
PL
FC
$N_{modules}$: 7
$Modularity$: 0.60
Efficiency:
1.17e-02
$N_{modules}$: 3
$Modularity$: 0.08
SC (^{10}log($N_{streams}$ + 1))
PL (mm)
FC (Pearson correlation)
Strength density (a.u., log scale)
SC ($N_{streams}$)
Length density (a.u.)
PL (mm)
Correlation density (a.u.)
FC (Pearson correlation)
Gamma (0.072)
Normal (0.015)
Density (a.u.)
Degree (SC matrix) [10^3]
Gamma (0.051)
Normal (0.066)
Closeness centrality (PL matrix)
Gamma (0.032)
Normal (0.065)
Degree (FC matrix)
156-Shen2013
SC
PL
FC
$N_{modules}$: 7
$Modularity$: 0.63
Efficiency:
1.11e-02
$N_{modules}$: 3
$Modularity$: 0.11
SC (^{10}log($N_{streams}$ + 1))
PL (mm)
FC (Pearson correlation)
Strength density (a.u., log scale)
SC ($N_{streams}$)
Length density (a.u.)
PL (mm)
Correlation density (a.u.)
FC (Pearson correlation)
Gamma (0.098)
Normal (0.026)
Density (a.u.)
Degree (SC matrix) [10^3]
Gamma (0.047)
Normal (0.061)
Closeness centrality (PL matrix)
Gamma (0.023)
Normal (0.082)
Degree (FC matrix)

Schaefer17Networks

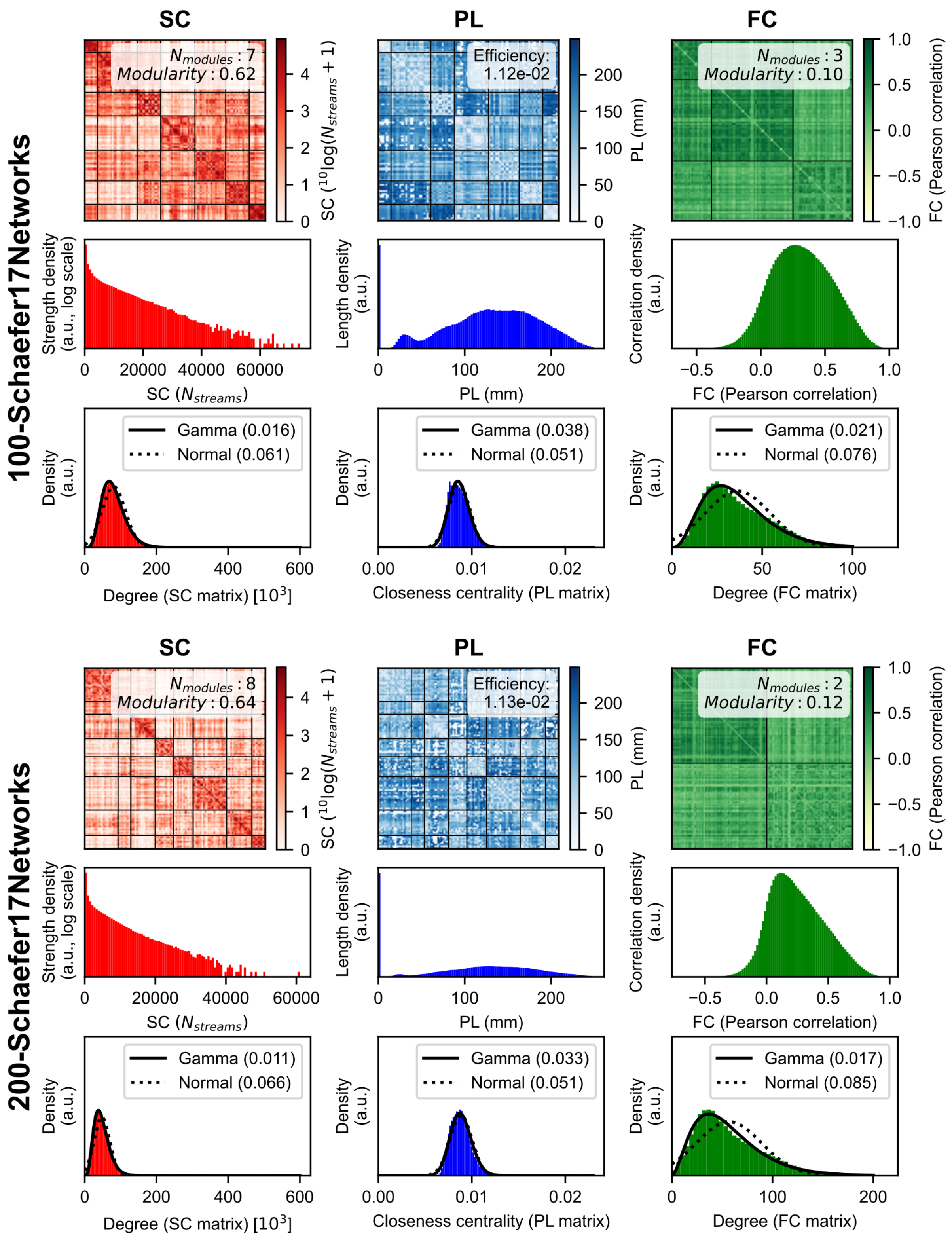

HarvardOxfordMaxProbThr0

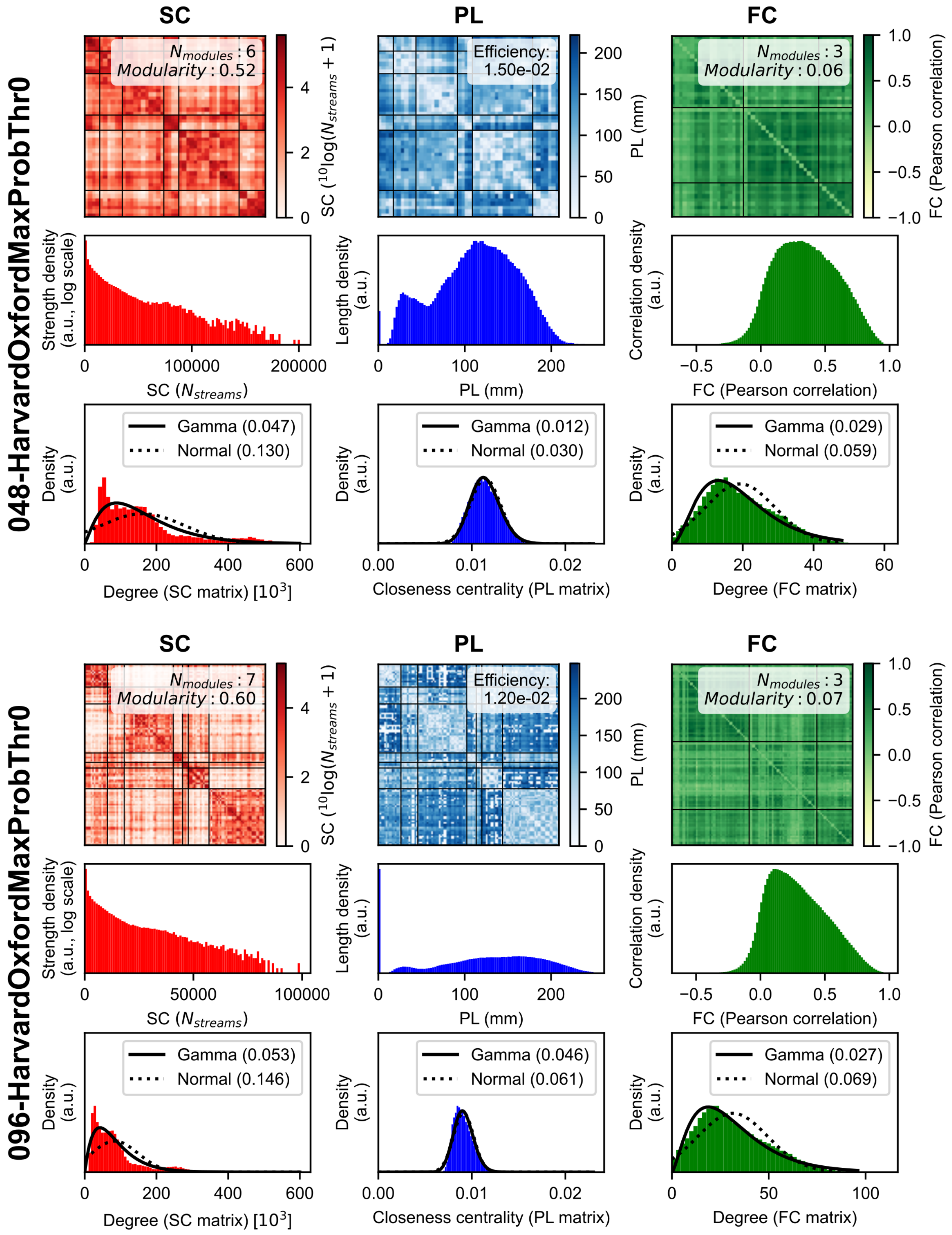

Structural atlases

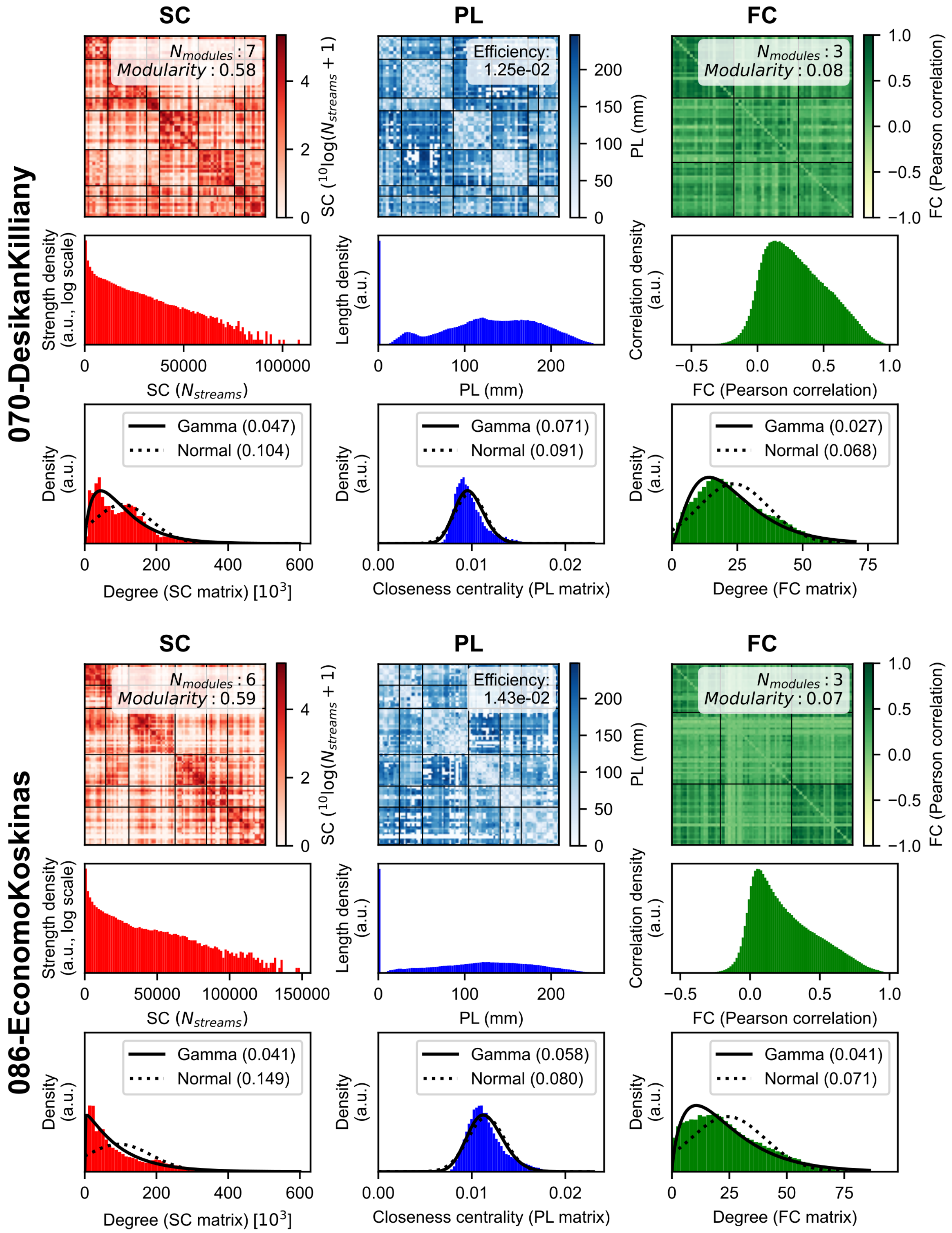

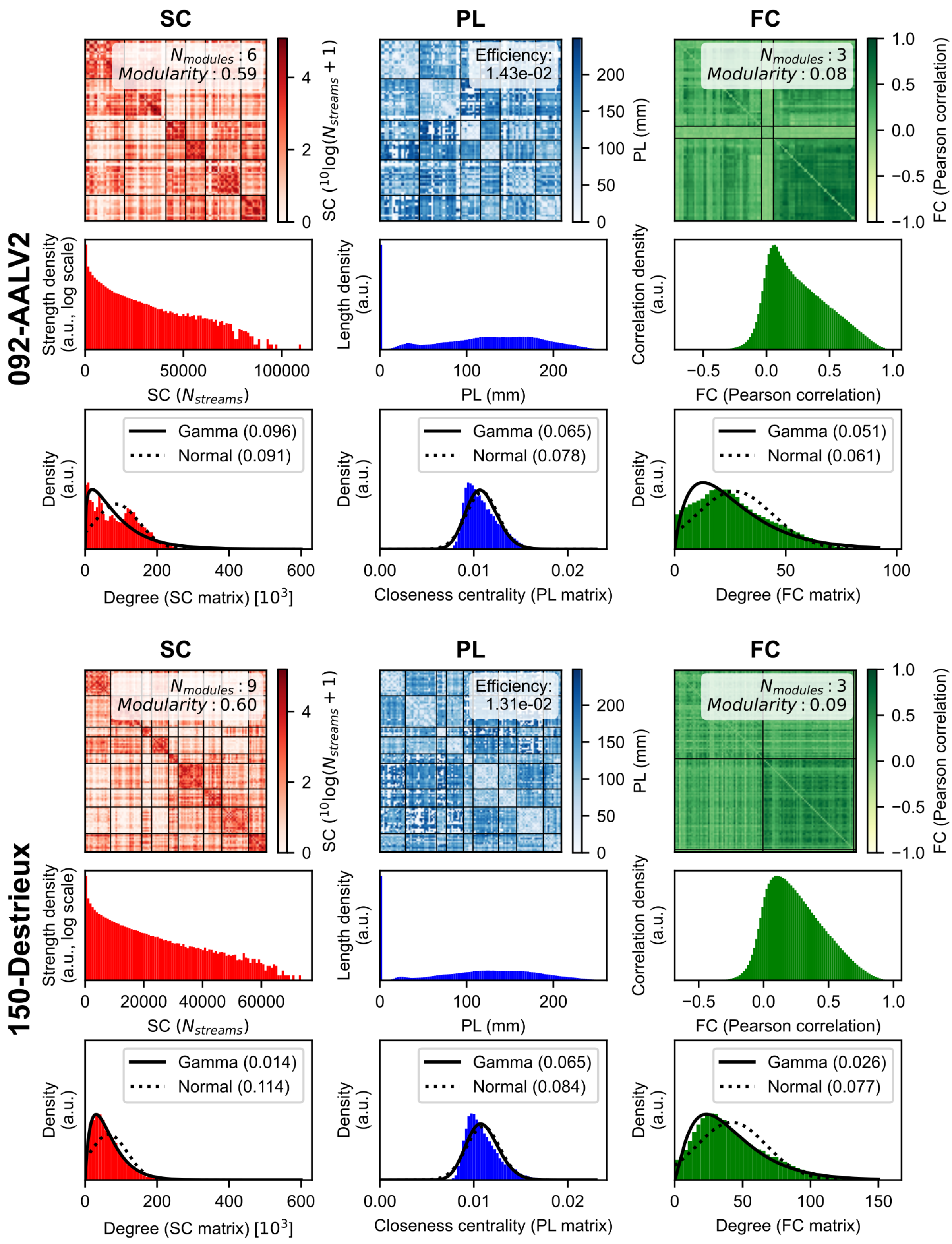
092-AALV2
150-Destrieux
SC
PL
FC
$N_{modules}: 6$
$Modularity: 0.59$
Efficiency:
1.43e-02
$N_{modules}: 3$
$Modularity: 0.08$
SC ($^{10}\log(N_{streams}+1)$)
PL (mm)
FC (Pearson correlation)
Strength density
(a.u., log scale)
SC ($N_{streams}$)
Length density
(a.u.)
PL (mm)
Correlation density
(a.u.)
FC (Pearson correlation)
Density
(a.u.)
Gamma (0.096)
Normal (0.091)
Degree (SC matrix) [10^3]
Gamma (0.065)
Normal (0.078)
Closeness centrality (PL matrix)
Gamma (0.051)
Normal (0.061)
Degree (FC matrix)
$N_{modules}: 9$
$Modularity: 0.60$
Efficiency:
1.31e-02
$N_{modules}: 3$
$Modularity: 0.09$
Gamma (0.014)
Normal (0.114)
Gamma (0.065)
Normal (0.084)
Gamma (0.026)
Normal (0.077)

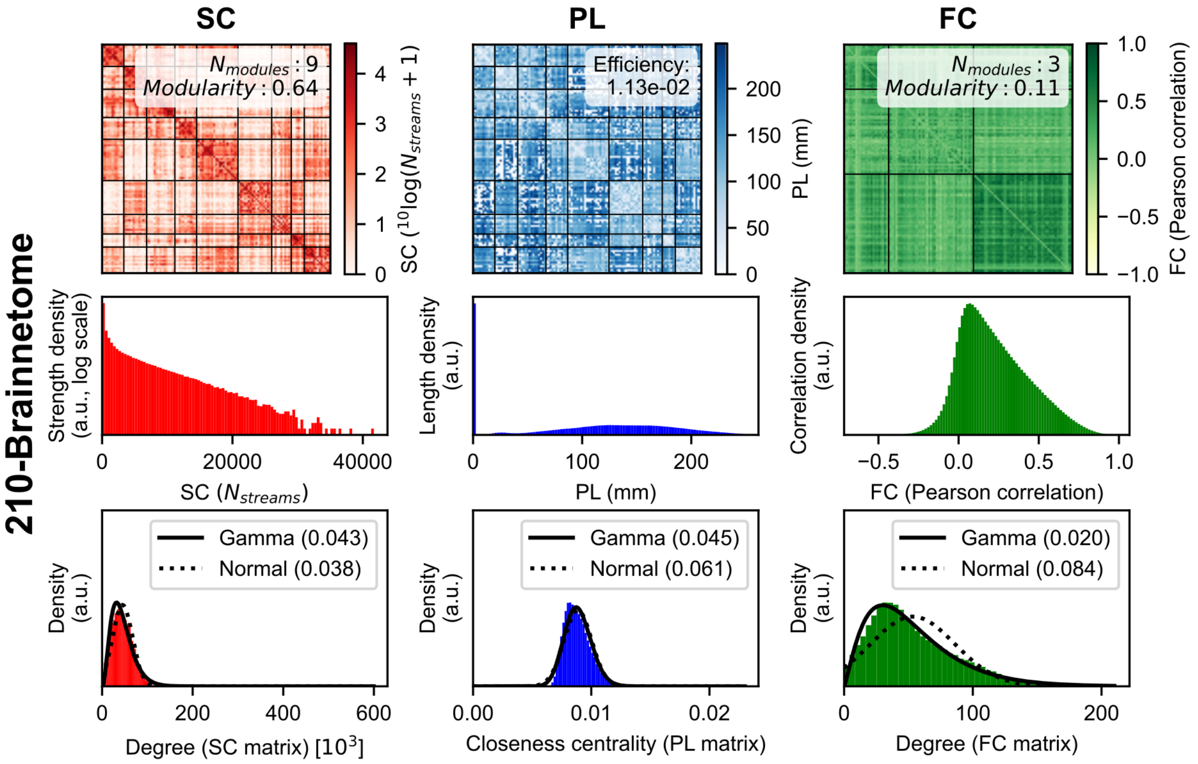

210-Brainnetome
SC
PL
FC
$N_{modules}$: 9
$Modularity$: 0.64
SC ($^{10}\log(N_{streams} + 1)$)
Efficiency:
1.13e-02
PL (mm)
$N_{modules}$: 3
$Modularity$: 0.11
FC (Pearson correlation)
Strength density
(a.u., log scale)
SC ($N_{streams}$)
Length density
(a.u.)
PL (mm)
Correlation density
(a.u.)
FC (Pearson correlation)
Density
(a.u.)
Gamma (0.043)
Normal (0.038)
Degree (SC matrix) [10^3]
Gamma (0.045)
Normal (0.061)
Closeness centrality (PL matrix)
Gamma (0.020)
Normal (0.084)
Degree (FC matrix)
