# Supplementary material for: Parcellation-induced variation of empirical and simulated brain connectomes at group and subject levels
Source: Netw Neurosci. 2021 Aug 30;5(3):798–830. doi: 10.1162/netn_a_00202 (PMC8567834; doi:10.1162/netn_a_00202)
Supplement: Supplementary file 2 [file netn-05-798-s002.pdf]

Domhof, J. W. M., Jung, K., Eickhoff, S. B. & Popovych, O. V. (2021). Supporting information for "Parcellation-induced variation of empirical and simulated brain connectomes at group and subject levels." *Network Neuroscience.* Advance publication. https://doi.org/10.1162/netn_a_00202

Supplementary Method

Table 1 includes the parcellation schemes that are compared with one another in this study. Here, we provide a summary on the construction of the parcellation schemes, i.e. what methodology was applied to what type of data to construct them. Additionally, we disclose how we modify each brain parcellation image for the purpose of enhancing the comparability between parcellations. These modifications typically comprise the following five steps:

- checking whether the image was sampled to MNI152 nonlinear template space,
- ensuring that its spatial resolution was 1 mm (resolution of dwMRI images in the HCP dataset) and that its coordinates were compliant with FSL standards,
- performing any transformations to warp the image to the MNI152 nonlinear template space,
- removing any cerebellar and subcortical regions and
- down-sampling the image to a 2 mm spatial resolution (resolution of the fMRI data in the HCP dataset).

In order to ease the procedures required in any of these steps, FreeSurfer's `recon_all` function is applied to acquire the tissue segmentation result corresponding to the MNI152 nonlinear template image. From the results of this tissue segmentation, we compile a grey matter mask spanning both hemispheres which we dilute with a 3 mm box kernel. Subsequently, we ensure that the diluted mask is compliant with FSL standards.

MIST atlas parcellations (Urchs et al., 2019)

In order to construct these whole-brain, volume-based parcellations, the functional connectivity data from 198 subjects was clustered using a hierarchical agglomerative clustering algorithm. In this study, we use the 36, 64, 122 and 197 parcels variants of the atlas. However, the comparability enhancing image modifications left respectively 31, 56, 103 and 167 parcels in the images. The details of these modifications are described below.

1. The parcellation images are in the MNI152 nonlinear template space.
2. The images have a resolution of 3 mm. To correct for this mismatch, we
    - reorient the images to FSL's standard orientation,
    - make a 3 mm resolution variant of the MNI152 nonlinear template image by down-sampling the 1 mm resolution original,
    - co-register the parcellation images to this 3 mm variant,
    - linearly transform the 3 mm resolution MNI152 nonlinear template image to its 1 mm resolution original and
    - warp the parcellation images to obtain their 1 mm variant.
3. As the parcellation images are now already in the appropriate MNI152 nonlinear template space, we are not required to perform any additional transformations.
4. The parcellation images contain cerebellar and subcortical regions, which are eliminated by multiplying the atlas images with the diluted grey matter mask (see above) and subsequently removing invaluable labels from the parcellation images; here, the total volume occupied by a parcel is required to be at least 500 voxels.
5. The parcellation images are down-sampled to a spatial resolution of 2 mm.

Craddock atlas parcellations (Craddock et al., 2012)

In order to construct these whole-brain, volume-based parcellations, the functional connectivity data from 41 subjects was clustered using a spectral clustering algorithm. We consider the parcellations that were realised by applying a two-level group clustering scheme to the spatial correlations between functional connectivity maps. We take the 40, 60, 120 and 180 parcels variants and modify them to enhance their comparability; respectively 38, 56, 108 and 160 parcels still remain in the parcellation images after these modifications. The details of these modifications are described below.

1. The parcellation images are in the MNI152 nonlinear template space.

2. The images have a resolution of 4 mm. To correct for this mismatch, we
    - first dilute the images with a 12 mm box kernel to account for the rather poor resolution,
    - reorient the images to FSL's standard orientation,
    - make a 4 mm resolution variant of the MNI152 nonlinear template image by down-sampling the 1 mm resolution original,
    - co-register the parcellation images to this 4 mm variant,
    - linearly transform the 4 mm resolution MNI152 nonlinear template image to its 1 mm resolution original and
    - warp the parcellation images to obtain their 1 mm variant.
3. As the parcellation images are now already in the appropriate MNI152 nonlinear template space, we are not required to perform any additional transformations.
4. The parcellation images contain cerebellar and subcortical regions, which are eliminated by multiplying the atlas images with the diluted grey matter mask (see above) and subsequently removing invaluable labels from the parcellation images; here, the total volume occupied by a parcel is required to be at least 500 voxels.
5. The parcellation images are down-sampled to a spatial resolution of 2 mm.

Shen 2013 atlas parcellations (Shen et al., 2013)

In order to construct these whole-brain, volume-based parcellations, the functional connectivity data from 79 subjects was clustered using a multigraph k-way clustering algorithm. In this study, we take the 100 and 200 parcels variants and modify them to enhance their comparability; respectively 79 and 156 parcels still remain in the parcellation images after these modifications. The details of these modifications are described below.

1. The parcellation images are in the MNI152 nonlinear template space.
2. The images have a resolution of 1 mm, but the coordinates and folding patterns are not consistent with the MNI152 nonlinear template. To correct for these mismatches, we
    - first dilute the images with a 3 mm box kernel to account for the incompatible folding patterns,
    - reorient the images to FSL's standard orientation,
    - co-register the parcellation images to the 1 mm MNI152 nonlinear template image.
3. As the parcellation images are now already in the appropriate MNI152 nonlinear template space, we are not required to perform any additional transformations.
4. The parcellation images contain cerebellar and subcortical regions, which are eliminated by multiplying the atlas images with the diluted grey matter mask (see above) and subsequently removing invaluable labels from the parcellation images; here, the total volume occupied by a parcel is required to be at least 500 voxels.
5. The parcellation images are down-sampled to a spatial resolution of 2 mm.

Schaefer atlas parcellations (Schaefer et al., 2018)

Voxels were grouped together by applying a gradient-weighted Markov Random Field to the functional connectivity data from 1489 subjects. The result was published as a cortical, surface-based atlas. In this study, we use the 100 and 200 parcels variants. Because the parcellations are surface-based, we sample these parcellations in the volumetric MNI152 nonlinear template space using the results from the `recon_all` function that was applied to the MNI152 nonlinear template image. The rest of the parcellation image modifications is described below.

1. The parcellation images are already in the right standard space, because the atlas is sampled through the use of the MNI152 nonlinear template image.
2. The spatial resolution is correct, but the coordinates are not consistent with FSL standards.The parcellation images are assigned the right coordinates through the use of the results from the `recon_all` function.

3. As the parcellation images are now already in the appropriate MNI152 nonlinear template space, we are not required to perform any additional transformations.
4. The parcellation images do not contain cerebellar or subcortical regions; still, the parcels are thinner than the other parcellation images and are therefore diluted with a 3 mm box kernel to ensure they cover a similar cortical volume.
5. The parcellation images are down-sampled to a spatial resolution of 2 mm.

Harvard-Oxford atlas parcellations (Desikan et al., 2006; Frazier et al., 2005; Goldstein et al., 2007; Makris et al., 2006)

In order to construct these volumetric parcellations, the cortical folding patterns of 37 subjects were analysed using semi-automated tools developed by the Harvard-Center for Morphometric Analysis. We use the maximum probability, 0% threshold, cortical variants with 48 (without hemispheric separation) and 96 (with hemispheric separation) parcels. The details of the modifications applied to these parcellations are listed below.

1. The parcellation images are in the MNI152 nonlinear template space.
2. The images have a resolution of 1 mm and their coordinates are consistent with the MNI152 nonlinear template.
3. The parcellation images are already in the appropriate MNI152 nonlinear template space.
4. The parcellation images do not contain cerebellar and subcortical regions. Still, parcels occupy a relatively large cortical volume. To correct for this, we multiply the parcellation images with the diluted grey matter mask (see above).
5. The parcellation images are down-sampled to a spatial resolution of 2 mm.

Desikan-Killiany atlas (Desikan et al., 2006)

The structural MRI data of 40 subjects were analysed by a registration procedure that aligns the cortical folding patterns to create this surface-based parcellation. Because the parcellation is surface-based, the same procedures as described for the Schaefer atlas are applicable here.

Von Economo-Koskinas atlas (Scholtens et al., 2018; von Economo & Koskinas, 1925)

20 human brains were analysed using histological tools in order to identify strong, spatial gradients regarding cytoarchitectonic properties. These gradients were then assumed to represent the boundaries of cortical regions. The original parcellation has been digitised and is available as a surface-based parcellation. Because the parcellation is surface-based, the same procedures as described for the Schaefer atlas are applicable here.

AAL atlas (version 2) (Rolls et al., 2015; Tzourio-Mazoyer et al., 2002)

The structural MRI data of 1 subject was analysed by a boundary detection algorithm called VoxeLine to create this volume-based parcellation. The original parcellation contains 120 parcels, but after we modified its image in order to enhance comparability only 92 cortical parcels remained. The details of these modifications are described below.

1. The parcellation image is not in the MNI152 nonlinear template space, but in the Colin27 linear template space.
2. As the atlas is not in the MNI152 nonlinear template space, checking its coordinates and resolution is irrelevant.
3. Since the atlas is not in the MNI152 nonlinear template space, it first has to be transformed to it. In order to do this we
   - reorient the parcellation and its original template image to FSL standards,
   - extract the brain from the original template image by multiplying it with its mask,
   - decrease the spatial resolution of the original template to 2 mm to ease the nonlinear transformation to the MNI152 nonlinear template space,
   - co-register the original template brain with the MNI152 nonlinear template brain image with 2 mm resolution,

    - nonlinearly transform the original template brain to the MNI152 nonlinear template brain image with 2mm resolution,
    - warp the parcellation image to the MNI152 nonlinear template space with 2 mm resolution,
    - co-register the MNI152 nonlinear 2 mm template brain with its 1 mm variant and, finally,
    - warp the parcellation image to the 1 mm MNI152 nonlinear template space.
4. The parcellation image contains cerebellar and subcortical regions, which are eliminated by multiplying the atlas images with the diluted grey matter mask (see above) and subsequently removing invaluable labels from the parcellation images; here, the total volume occupied by a parcel is required to be at least 500 voxels.
5. The parcellation images are down-sampled to a spatial resolution of 2 mm.

Destrieux atlas (Destrieux et al., 2010)

The structural MRI data of 12 subjects was analysed in order to divide the brain into gyral and sulcal regions. This division was performed through a consideration of surface curvature and convexity values as well as prior labelling probabilities and neighbouring labels. The parcellation is surface-based and therefore the same procedures as described for the Schaefer atlas are applicable here.

Brainnetome atlas (Fan et al., 2016)

The structural connectivity data from 40 subjects was clustered using a spectral clustering algorithm to create this parcellation. We use the surface-based cortical version of the parcellation. Because the parcellation is surface-based, the same procedures as described for the Schaefer atlas are applicable here.
