# Supplementary material for: Parcellation-induced variation of empirical and simulated brain connectomes at group and subject levels
Source: Netw Neurosci. 2021 Aug 30;5(3):798–830. doi: 10.1162/netn_a_00202 (PMC8567834; doi:10.1162/netn_a_00202)
Supplement: Supplementary file 3 [file netn-05-798-s003.pdf]

Domhof, J. W. M., Jung, K., Eickhoff, S. B. & Popovych, O. V. (2021). Supporting information for "Parcellation-induced variation of empirical and simulated brain connectomes at group and subject levels." *Network Neuroscience*. Advance publication. https://doi.org/10.1162/netn_a_00202

Supplementary Results

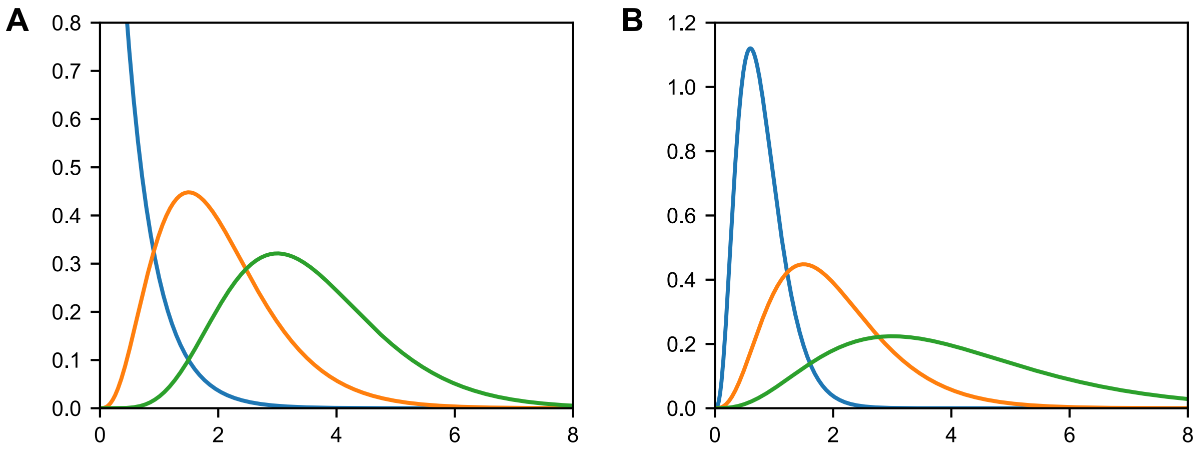

Fig. S1. **(A)** Gamma probability distribution function for fixed scale parameter $\theta = 0.5$ and varying shape parameter k. Blue, orange and green correspond to $k = 1$, $k = 4$ and $k = 7$, respectively. **(B)** Gamma probability distribution function for fixed shape parameter $k = 4$ and varying scale parameter θ. Blue, orange and green correspond to $\theta = 0.2$, $\theta = 0.5$ and $\theta = 1.0$, respectively.

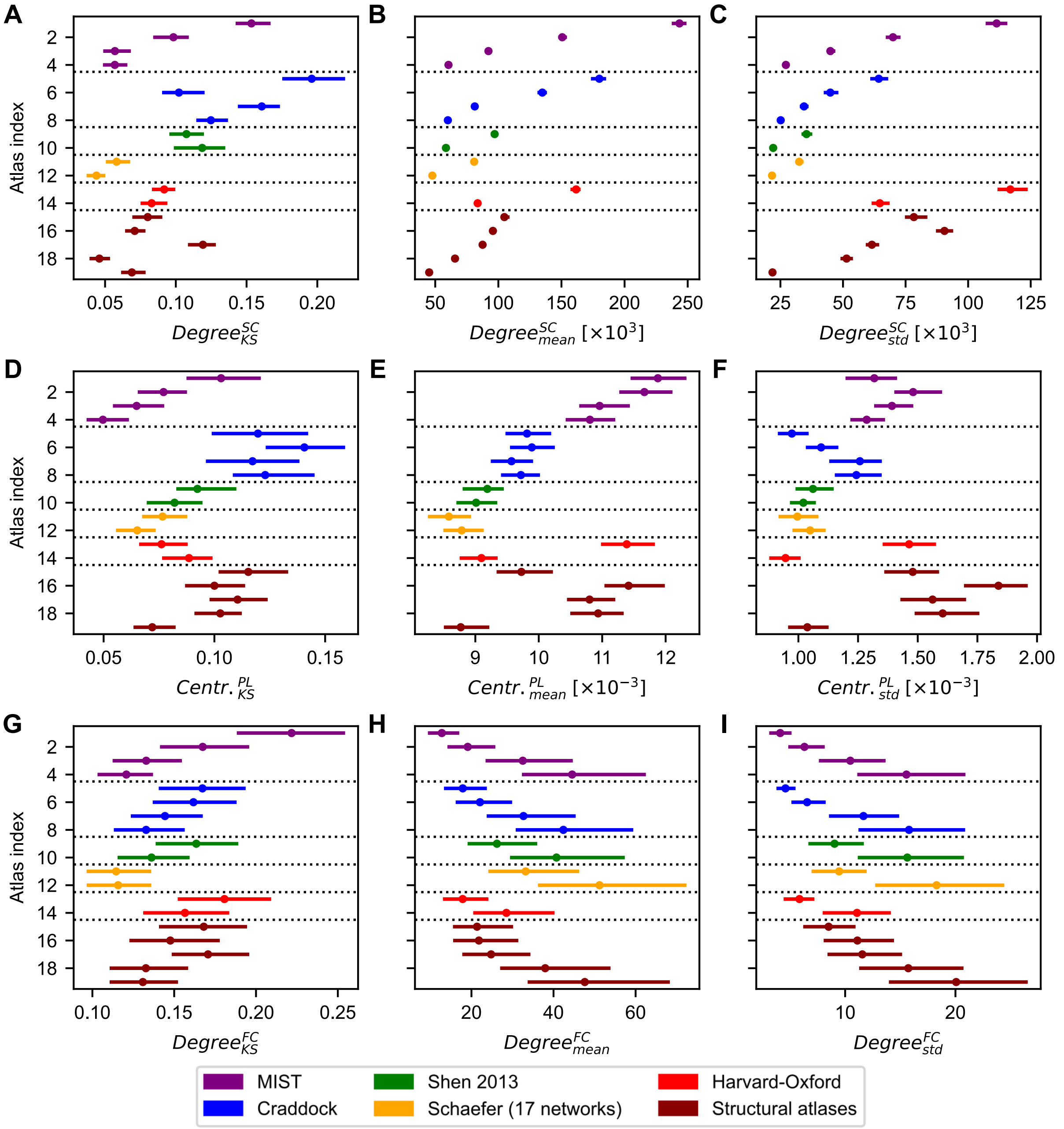

Fig. S2. (A-C) Kolmogorov-Smirnov (KS) statistics of the fit of the gamma distribution to the empirical degree distribution of the structural connectivity (SC) (A) and the mean (B) and the standard deviation (C) of that type of degree. **(D-F)** Same quantities as in panel A to C but for the closeness centrality of the path length (PL) matrix. **(G-I)** Same quantities as in panel A to C but for the degree of the functional connectivity (FC) matrix. Dots and lines depict the medians and interquartile ranges across subjects, respectively, and the atlas indices on the vertical axes correspond to those in Table 1 which contains the information about the used parcellations. Abbreviations: centr. = closeness centrality.

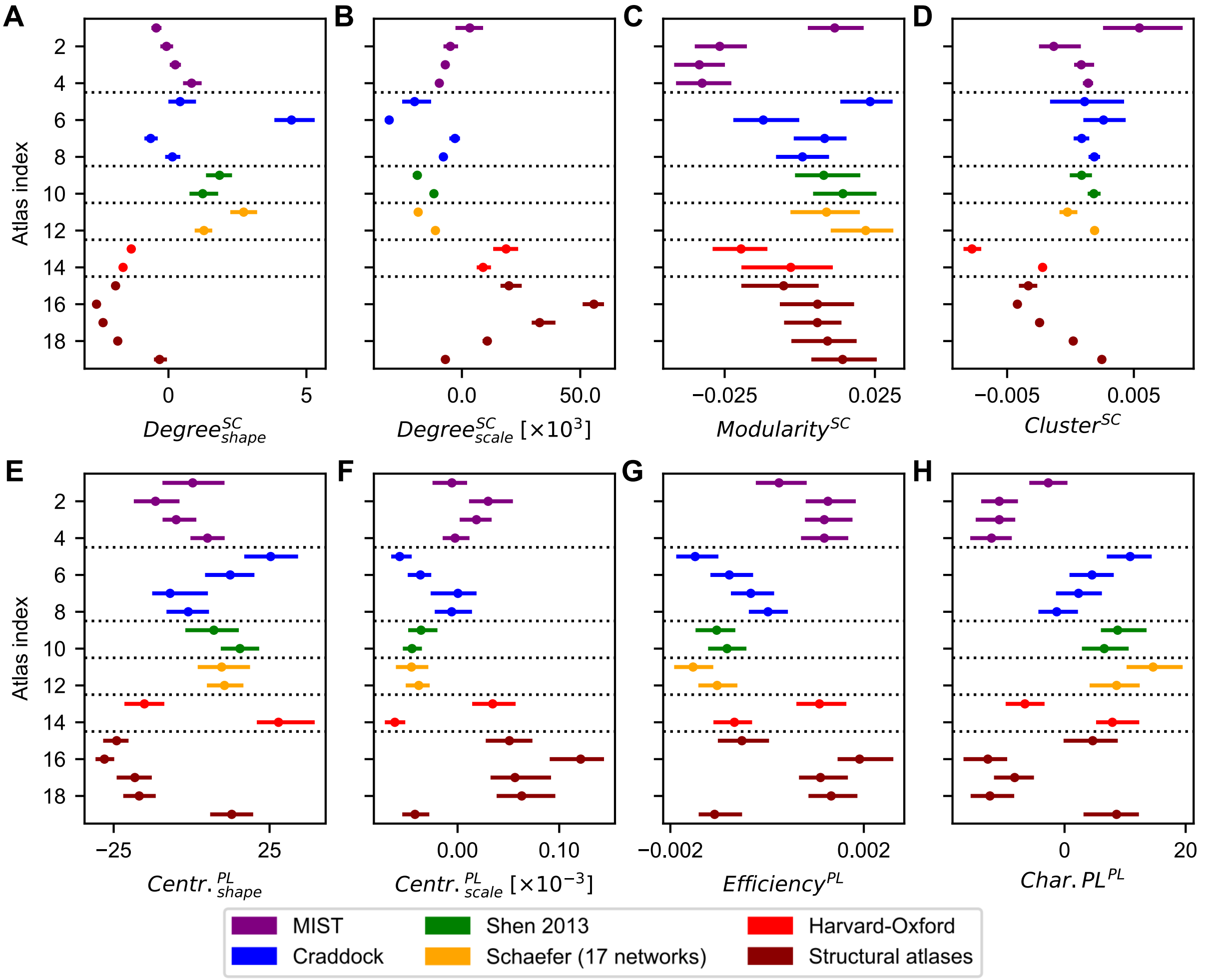

Fig. S3. (A-D) Statistics extracted from the structural connectivity (SC) matrices, which are the shape (A) and scale (B) parameters of the degree distributions, the modularities (C) and the clustering coefficients (D). **(E-H)** Statistics extracted from the path length (PL) matrices, which are the shape (E) and scale (F) parameters of the closeness centrality distributions, the global efficiencies (G) and the characteristic path lengths (H). Dots and lines depict the medians and interquartile ranges across subjects, respectively. The atlas indices on the vertical axes correspond to those in Table 1 which contains the information about the used parcellations. The difference between these plots and those shown in Fig. 2 is that here the effect of granularity has been regressed out. Abbreviations: Centr. = closeness centrality, Char.PL = characteristic path length.

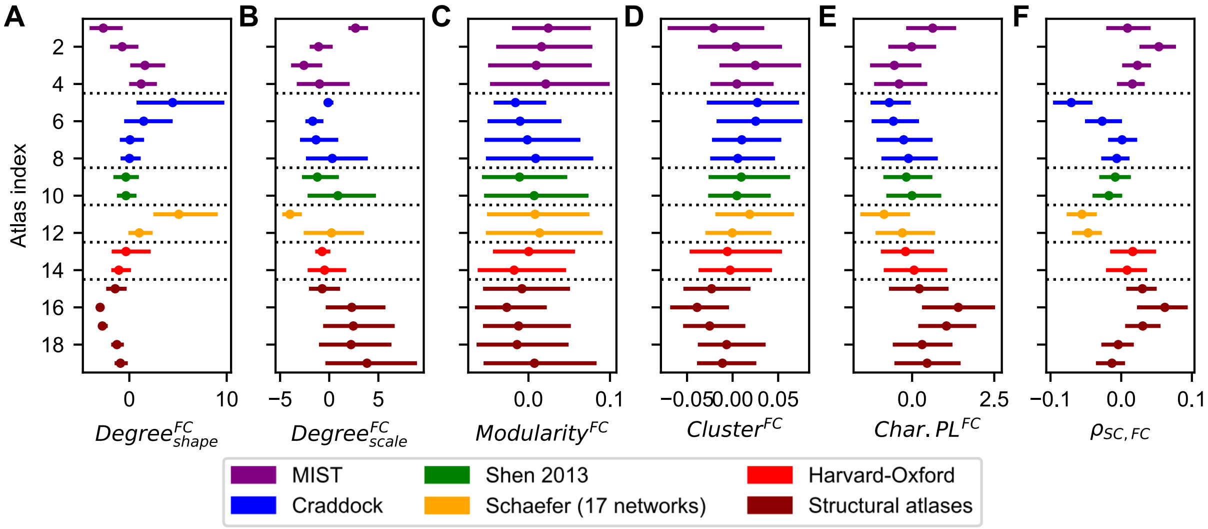

Fig. S4. (A-E) Statistics extracted from the empirical FC matrices, which are the shape (A) and scale (B) parameters of their degree distributions, their modularities (C), their clustering coefficients (D) and their characteristic path lengths (E). **(F)** Pearson correlation coefficients corresponding to the structure-function relationship between the upper triangular parts (excluding diagonal) of the empirical SC and FC matrices. Dots and lines depict the medians and interquartile ranges across subjects, respectively. The atlas indices on the vertical axes correspond to those in Table 1 which contains the information about the used parcellations. The difference between these plots and those shown in Fig. 3 is that here the effect of granularity has been regressed out. Abbreviations: Char.PL = characteristic path length.

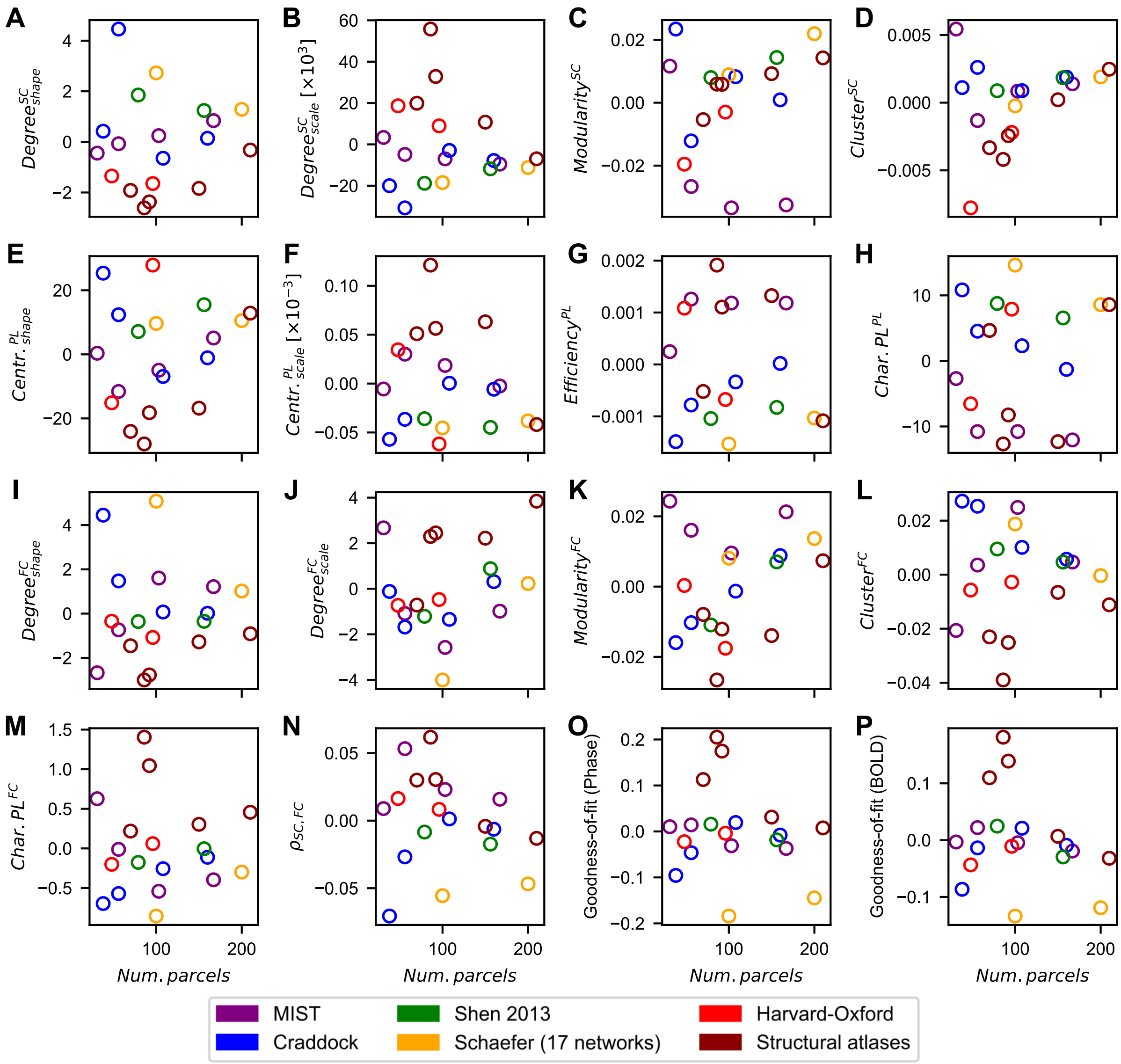

Fig. S5. Same as Fig. 4, though with the difference that in these plots the measures are plotted as functions of the number of parcels instead of its inverse and that the granularity effects displayed in Fig. 4 have been regressed out. Each dot corresponds to a particular atlas. Abbreviations: Centr. = closeness centrality, Char.PL = characteristic path length.

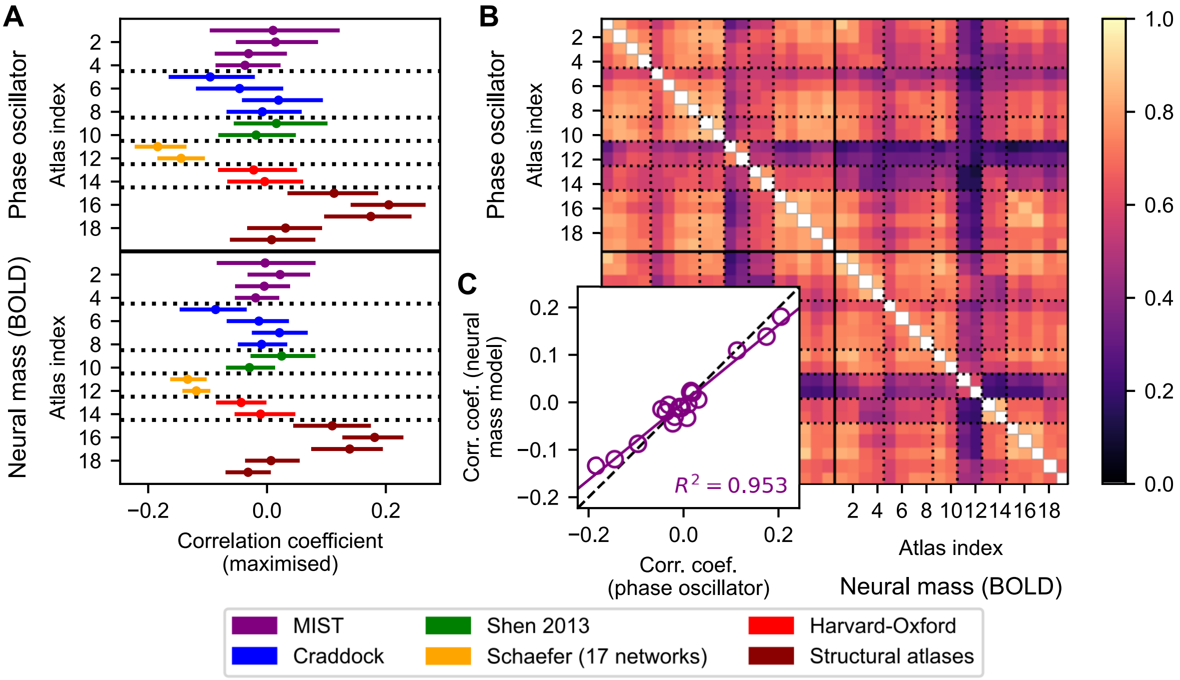

Fig. S6. (A) Maximised correlations (goodness-of-fit) between the empirical and simulated FC matrices for the brain parcellation schemes and models investigated in this study as indicated on the vertical axes. Dots and lines depict the medians and interquartile ranges across subjects, respectively. **(B)** Correlations across subjects of the goodness-of-fit of the model between the considered parcellations and models. Table 1 contains the parcellation information corresponding to the atlas indices used in the plots. **(C)** Scatter plot of the medians of the goodness-of-fit corresponding to the phase oscillator (x-axis) and neural mass model (y-axis) across subjects. Each dot corresponds to a particular parcellation, the purple line portrays the linear regression between both types of goodness-of-fit and the black dashed line corresponds to $x = y$. The difference between these plots and those shown in Fig. 6 is that here the effect of granularity has been regressed out.

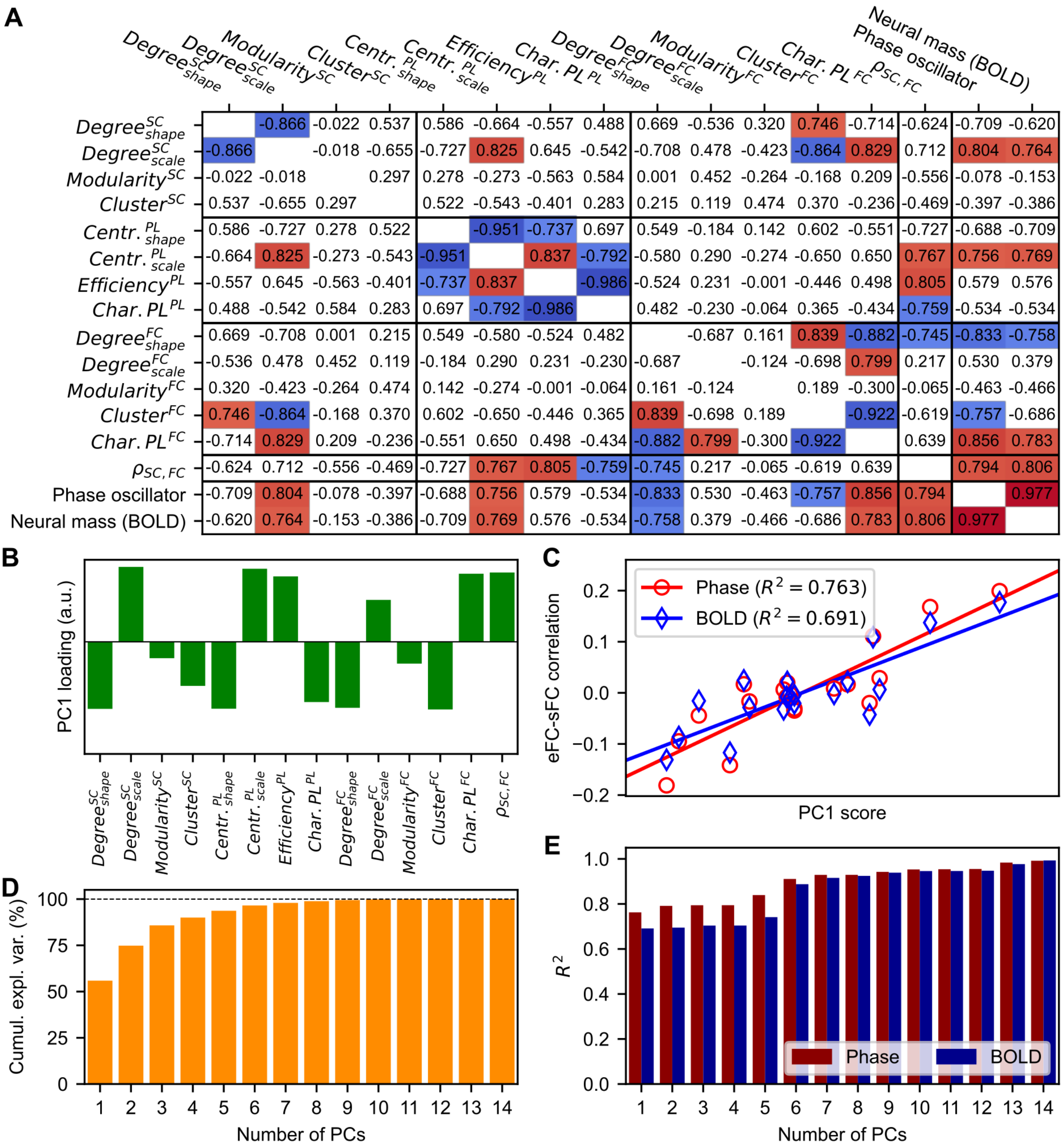

Fig. S7. **(A)** Cross-correlations among the inverted granularities, the graph-theoretical measures of the empirical connectomes (network properties depicted in Fig. 2 and Fig. 3), the structure-function relationship and the goodness-of-fit of the models to the empirical data. The correlation was calculated across parcellations between the median values over all subjects. Significant correlations are highlighted by colours (p<0.05, two-sided, Bonferroni corrected). **(B)** Loadings of the first (PC1) and the second (PC2) principal components of the group-averaged graph-theoretical metrics, i.e. the contributions of the original empirical data variables to PC1 and PC2. **(C)** Regressions of the PC1 scores with the medians of the goodness-of-fit between empirical (eFC) and simulated (sFC) functional connectivity. The medians were calculated across subjects for each considered parcellation for the phase oscillator (red) and the neural mass model (blue) as indicated in the legend together with the fraction of the explained variance. The symbols stand for the individual parcellations from Table 1. **(D)** Cumulative amount of explained variance in the group-averaged graph-theoretical measures as a function of the number of included PCs. **(E)** Fraction of the interparcellation variance of the goodness-of-fit being explained by the (multivariate) linear regression model as a function of the number of PCs included in the model. The difference between these plots and those shown in Fig. 7is that here the effect of granularity has been regressed out. Other abbreviation: a.u. = arbitrary unit, cumul. = cumulative, expl. = explained, var. = variance.

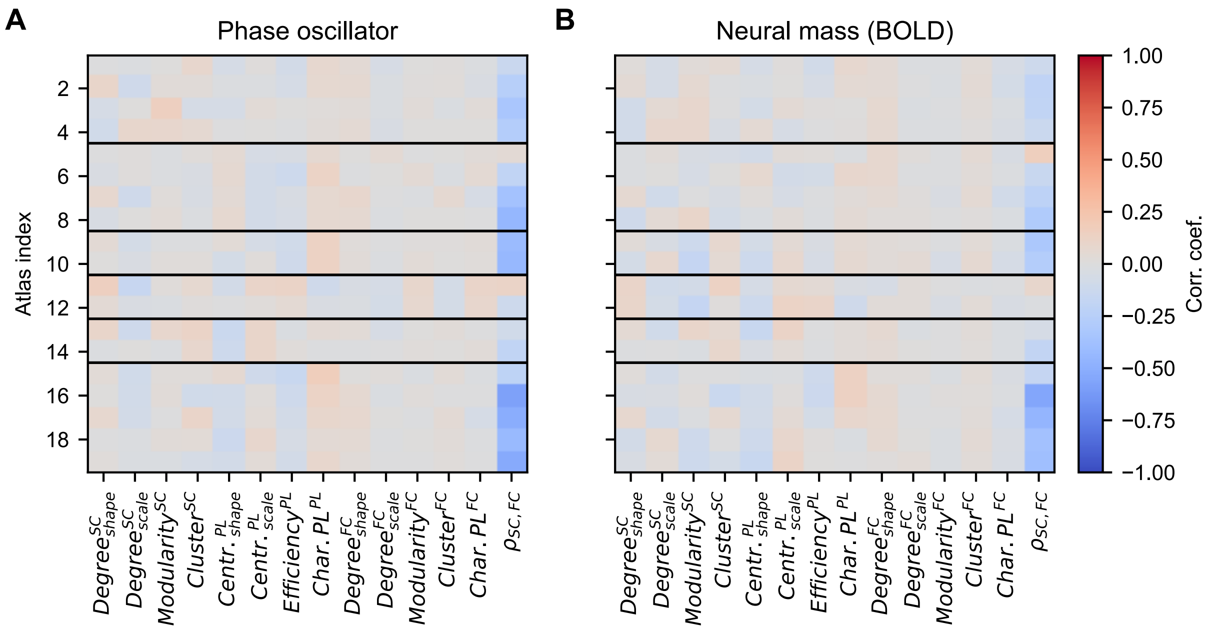

Fig. S8. Pearson correlation coefficients between the goodness-of-fit and the empirical data variables shown in Fig. 2 and Fig. 3 across subjects per parcellation for the phase oscillator (A) and the electrical model (B). Table 1 contains the parcellation information corresponding to the atlas indices used in the plots. Abbreviations: coef. = coefficient, corr. = correlation.

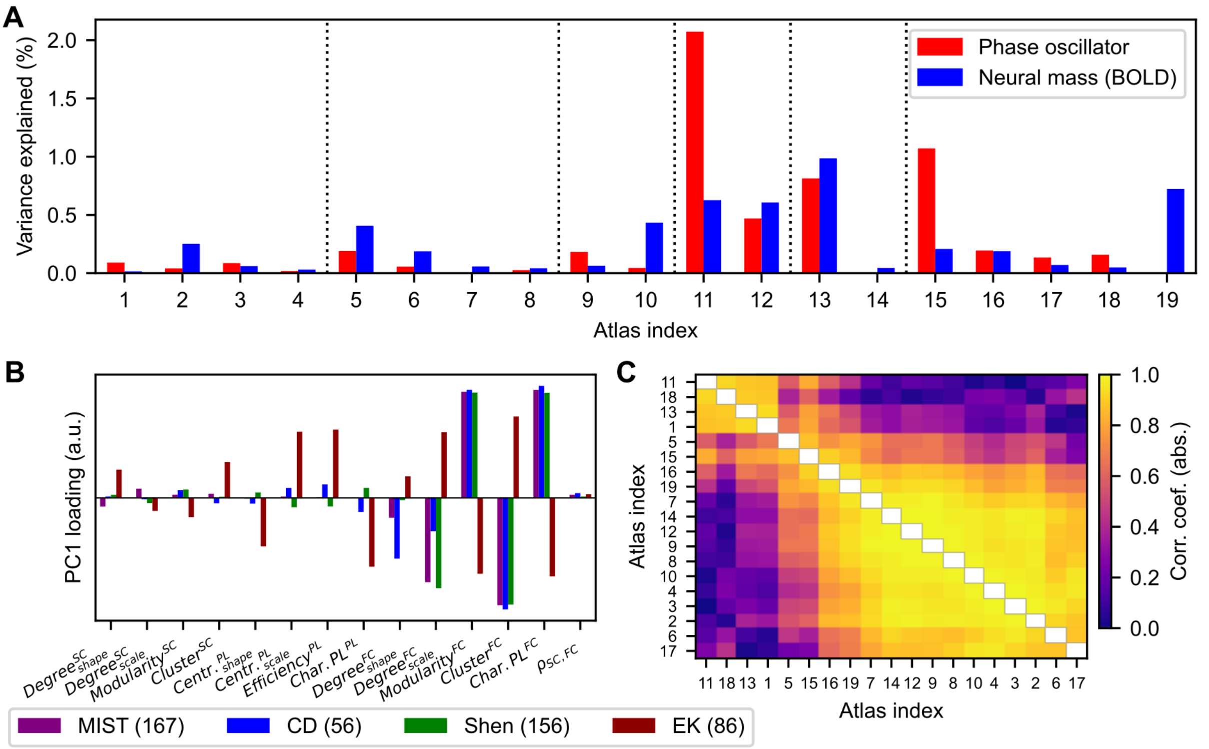

Fig. S9. (A) Amounts of within-parcellation, between-subject variance in the modelling results being explained by the combination of PCA with univariate, ordinary least squares linear regression (same approach as in Fig. 7B-E) per parcellation. Modelling results are sampled by using the coupled phase oscillators (red) and neural mass models (blue) and comprise the maximised correlation coefficients between the upper triangles of the empirical and simulated functional connectivity matrices excluding the diagonals. **(B)** Loadings of the first principal component (PC1) corresponding to the data variables depicted in Fig. 2 and Fig. 3 for a selection of 4 brain parcellation schemes. The abbreviations ”Shen (79)”, ”Sch. (100)”, ”HO (96)” and ”EK (86)” correspond to the parcellations in Table 1 with indices 9, 11, 14 and 16, respectively. **(C)** Absolute values of the Pearson correlation coefficients across the loadings per pair of brain parcellation. Table 1 contains the parcellation information corresponding to the atlas indices used in the plots. Other abbreviations: abs. = absolute value, coef. = coefficient, corr. = correlation.

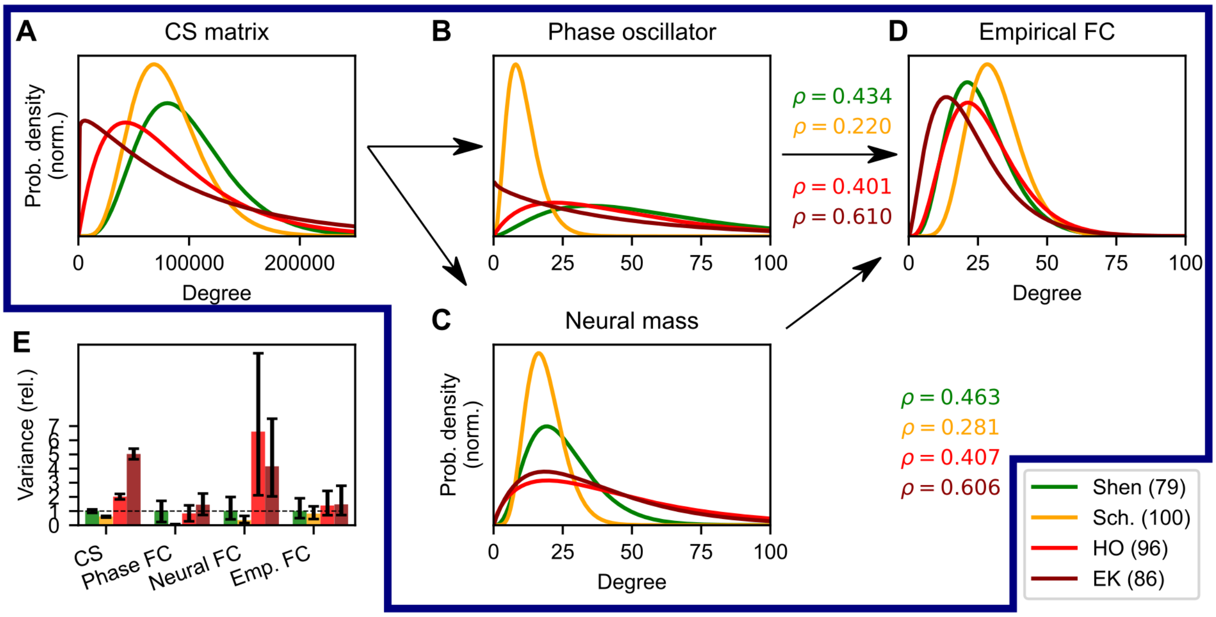

Fig. S10. (A) Characteristic degree distributions of the connection strength (CS) matrices for a selection of 4 parcellation schemes. **(B-D)** Same as panel A but here the empirical (emp. FC, D) and simulated functional connectivity matrices corresponding to the phase oscillator (phase FC, B) and neural mass (neural FC, C) models that provided the best fit are considered. Distributions are shown as normalised probability density functions and are constructed using the medians of their corresponding parameters across subjects. Coloured texts denote the median maximised correlation coefficients. **(E)** Relative variances included in the degree distributions. Bars and errorbars depict the medians and interquartile ranges across subjects. The abbreviations ”Shen (79)”, ”Sch. (100)”, ”HO (96)” and ”EK (86)” correspond to the parcellations in Table 1 with indices 9, 11, 14 and 16, respectively.
